# Transcription Factor Binding Site Analysis Identifies FOXO Transcription Factors as Regulators of the Cutaneous Wound Healing Process

**DOI:** 10.1371/journal.pone.0089274

**Published:** 2014-02-19

**Authors:** Karl Markus Roupé, Srinivas Veerla, Joshua Olson, Erica L. Stone, Ole E. Sørensen, Stephen M. Hedrick, Victor Nizet

**Affiliations:** 1 Department of Pediatrics, University of California San Diego, La Jolla, California, United States of America; 2 Division of Oncology, Department of Clinical Sciences, Lund University and Lund University Hospital, Lund, Sweden; 3 Molecular Biology Section, Division of Biological Sciences, University of California San Diego, San Diego, California, United States of America; 4 Department of Cellular and Molecular Medicine, University of California San Diego, San Diego, California, United States of America; 5 Division of Infection Medicine, Department of Clinical Sciences, Lund University, Lund, Sweden; 6 Skaggs School of Pharmacy and Pharmaceutical Sciences, University of California San Diego, La Jolla, California, United States of America; National Research Council of Italy, Italy

## Abstract

The search for significantly overrepresented and co-occurring transcription factor binding sites in the promoter regions of the most differentially expressed genes in microarray data sets could be a powerful approach for finding key regulators of complex biological processes. To test this concept, two previously published independent data sets on wounded human epidermis were re-analyzed. The presence of co-occurring transcription factor binding sites for FOXO1, FOXO3 and FOXO4 in the majority of the promoter regions of the most significantly differentially expressed genes between non-wounded and wounded epidermis implied an important role for FOXO transcription factors during wound healing. Expression levels of FOXO transcription factors during wound healing *in vivo* in both human and mouse skin were analyzed and a decrease for all FOXOs in human wounded skin was observed, with FOXO3 having the highest expression level in non wounded skin. Impaired re-epithelialization was found in cultures of primary human keratinocytes expressing a constitutively active variant of FOXO3. Conversely knockdown of FOXO3 in keratinocytes had the opposite effect and in an *in vivo* mouse model with FOXO3 knockout mice we detected significantly accelerated wound healing. This article illustrates that the proposed approach is a viable method for identifying important regulators of complex biological processes using *in vivo* samples. FOXO3 has not previously been implicated as an important regulator of wound healing and its exact function in this process calls for further investigation.

## Introduction

It is increasingly recognized that stable clusters of co-occurring transcription factor binding sites (TFBS) coordinately regulate gene sets associated with highly specific cellular activities [1]–[3]. We hypothesized that a search for significant enrichment of TFBS, located in close proximity to one another in the promoter regions of the most differentially expressed genes in genome wide microarray data set, would therefore represent a powerful approach to find key regulators of a complex biological process. We tested this hypothesis by re-analyzing two published data sets on the human epidermal response to injury using the TFBS analysis program, Systematic Motif Analysis Retrieval Tool (SMART). The SMART software has previously been demonstrated to faithfully reproduce ChIP on Chip analysis results using this approach [1], [4].

Rapid re-establishment of epidermal barrier function in response to injury is critically important to prevent infections and formation of chronic wounds. Re-epithelialization involves keratinocyte migration and proliferation, and the epidermis must recruit and direct both the innate and the adaptive immune system during wound healing [5]–[7]. Consequently, significant efforts have been directed at understanding the epidermal response to injury including the use of gene expression profiling arrays, an especially powerful approach for understanding complex biological processes [8]–[13]. Nevertheless, pathways underlying human cutaneous wound healing are still poorly defined. The combined presence of resident dermal cells and infiltrating inflammatory cells in some of the studies have made it difficult to delineate important pathways and attribute specific roles to keratinocytes.

Here we chose two published genome wide microarray studies on isolated human epidermis for re-analysis [14], [15]. Using the now freely available TFBS analysis program SMART developed by one of the co-authors, we found an overrepresentation of TFBS for FOXO1, FOXO3 and FOXO4 in the most differentially expressed genes in both data sets. The program furthermore determined that FOXO1, FOXO3 and FOXO4 TFBS were positioned in close proximity to one other for the majority of these genes. Forkhead box “O” (FOXO) transcription factors, whose activity is regulated post-translationally [16], [17], have previously been studied for their roles in the cell cycle regulation, programmed cell death, longevity, DNA repair, vascular development, reactive oxygen species detoxification pathways, and regulation of adaptive and innate immune responses [18]–[22]. In corroborative experimental studies to validate the *in silico* analysis, we found that FOXO levels dropped during wound healing. Furthermore, the expression of a constitutively active variant of FOXO3 delayed keratinocyte scratch closure, whereas a knockdown of FOXO3 had the opposite effect. In addition deletion of FOXO3 led to accelerated wound healing in an *in vivo* mouse model. Our study illustrates the possibility of identifying important transcription factors controlling complex biological processes through re-analysis of previously published data sets using the SMART algorithm, in this case implicating FOXO3 as a potential key regulator of the cutaneous wound healing process.

## Methods

### Bacteria, cells and adenoviral vectors

The human group A *Streptococcus* serotype M1T1 isolate, 5448 used, was originally isolated from a patient with necrotizing fasciitis and toxic shock [23] and has been previously characterized [24]. Bacteria were propagated at 37°C on Todd-Hewitt agar (THA) (Difco) or in static liquid cultures of Todd-Hewitt broth (THB). Primary human keratinocytes were purchased from Lonza (Basel, Switzerland) and were cultured in KBM medium with KGM-2 growth supplements (Lonza) unless otherwise mentioned. Adenoviral vectors Ad-CMV-FKHRL1 (FOXO3 AAA) and Ad-GFP (GFP control) were obtained from Vector Biolabs (Philadelphia, PA).

### Mice

The FOXO3 knockout mice were originally characterized in the group of Prof. Karen Arden [25]. FOXO3f/f mice [26] were crossed with LysMCre transgenic mice [27] from Jackson Labs to get FOXO3 LysMCre knockout mice. CD1 mice and age matched C57BL/6J control mice for FOXO3 and FOXO3 LysMCre mice were from Jackson laboratories. All animal protocols used in this study were approved by the Animal Subjects Program of the University of California at San Diego and conformed to National Institute of Health guidelines and public law. All mice were maintained on a 12 h light/dark cycle with food and water provided *ad libitum*.

### Data analysis

**Figure 1:**
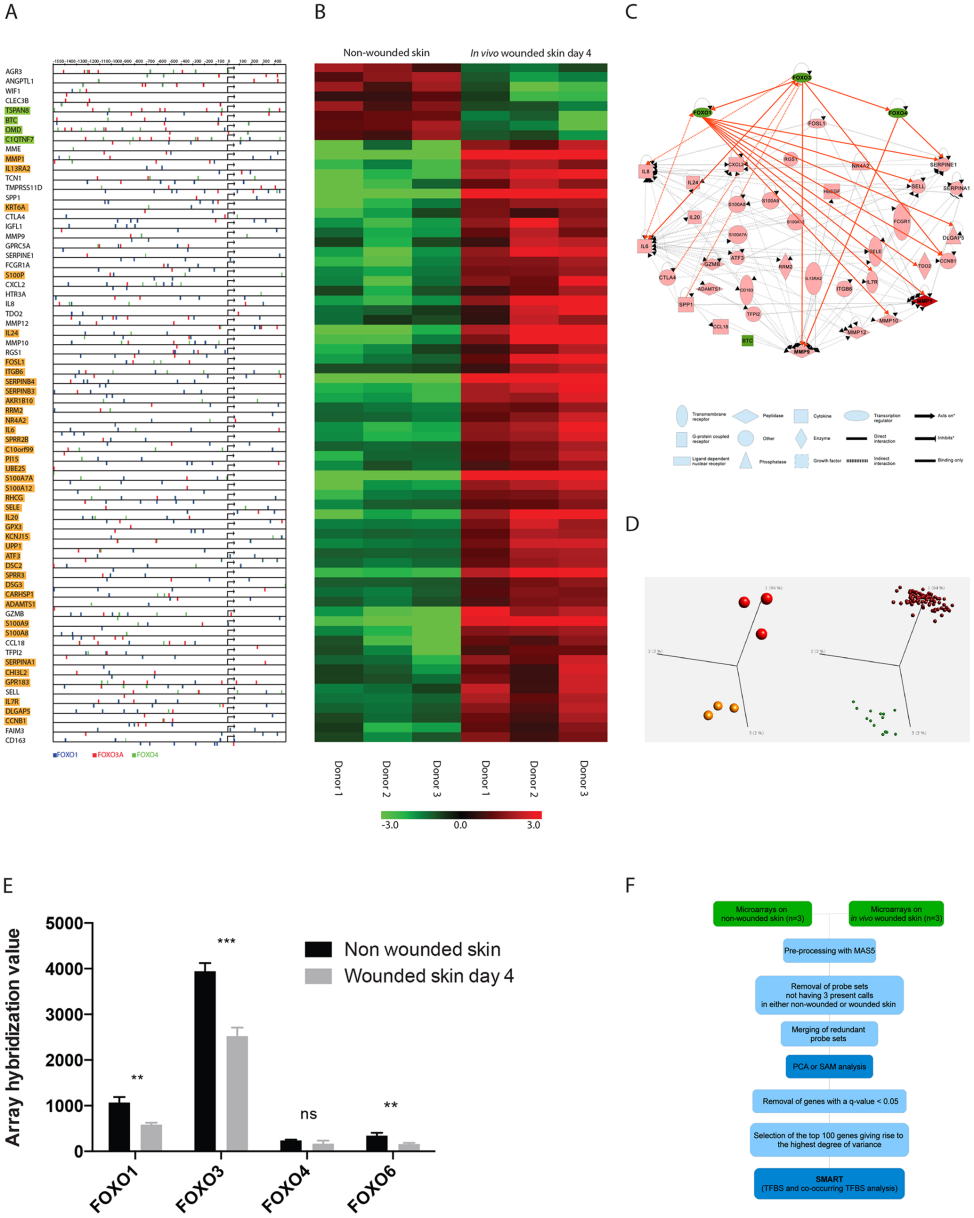
Re-analysis of a microarray data set from [14] on non wounded and *in vivo* wounded human skin samples. A) The promoter sequences for the 100 most differentially expressed genes between wounded and non-wounded skin were probed for transcription factor binding sites and co-occurring transcription factor binding sites. The presence of co-occurring transcription factor binding sites of FOXO1, FOXO3 and FOXO4 found by the SMART software to be within 50 base pairs of each other is depicted in the promoter regions of the 70 genes out of 100 were they were found. The presence of co-occurring transcription factor binding sites of either FOXO1, FOXO3 or FOXO4 in these promoter regions are also depicted. Some of these sites are partially overlapping. Genes with a Pavlidis template matching correlation coefficient of 0.9 to FOXO3 expression are highlighted; yellow equals positive correlation and green negative correlation B) Hierarchical clustering and heat map of the 70 genes containing co-occurring FOXO1-FOXO3-FOXO4 transcription factor binding sites. C) Schematic of currently known and annotated interactions between the 70 genes containing co-occurring FOXO1-FOXO3-FOXO4 binding sites generated by ingenuity pathway analysis software. FOXO1, FOXO3 and FOXO4 were also added to visualize how they interact with the selected genes. Only genes with at least one known connection to FOXO1, FOXO3, FOXO4, or one of the other 70 genes were included. D) PCA plots of the 100 selected genes depicting the remaining variance between the samples on the left and a synchronized PCA plot of the variables (gene expression) giving rise to the variance on the right. Genes were selected by first removing all genes having a q-value>0.05 based on a two group comparison analysis between wounded and non-wounded samples. Remaining genes were then filtered by variance till the top 100 genes remained with a p-value≤0.008. The selected genes stood for 23% of the total amount of variance in the data set. E) The normalized hybridization levels of FOXO transcription factor transcripts from the micro array data set from Roupé et. al. 2010 are depicted. A two-tailed Student's t-test confirmed a differential expression of FOXO1, FOXO3 and FOXO6 when comparing non-wounded and *in vivo* wounded human skin (*P<0.05, **P<0.01, ***P<0.001). Error bar denotes mean ±SD (n = 3). F) Flowchart giving an overview over the analysis approach (see methods section for more details.)

Raw data files from the previously published data set from Roupé et al 2010 [28] with array express accession number E-MEXP-3305 were completely reanalyzed (see **fig. 1f** for overview). Data were preprocessed and normalized using the MAS5 algorithm. Non-annotated probe sets and probe sets not having three present calls in either the non-wounded control or in the *in vivo* wounded condition were excluded. Redundant probe sets were merged together by taking mean values, resulting in a list of unique annotated genes. These were imported into the trial version of Qlucore principal component analysis (PCA). A matched two-group comparison test was first conducted, and only genes with a q-value≤0.05 were selected for further analysis. Of these, the top 100 genes giving rise to the highest degree of variance between non-wounded and *in vivo* wounded human epidermis were selected with a p-value≤0.008. Together, these genes contributed to 23.5% of the total variance between the two groups; a Significant Analysis of Microarrays (SAM) was used to verify the results generated by the PCA analysis. To further validate the robustness of subsequent analysis, lists of the top 150 and top 200 genes were selected. These lists of genes and the most differentially expressed 100 genes from Kennedy-Crispin et al [15] were subsequently analyzed by a contemporary version of the transcription factor binding site analyzes program SMART as previously described [1], [4]. Briefly the promoter regions 1500 base pairs upstream and 500 base pairs downstream of the genes from the two lists were scanned for enriched TFBS and whether these enriched TFBS were co-occurring within 50 base pairs from each other. The likelihood of randomly finding the resulting number of each TFBS was then assessed by comparing it to the number of found TFBS in the promoter regions from 10^5^ gene lists of the same sample size. These lists were generated by randomly sampling an equivalent number of genes from the human genome.

### *In vivo* wounds in murine skin

Mice between 8–12 weeks were used for *in vivo* wound experiments. Animals were anaesthetized with isoflorane, and a dorsal area of the skin was shaved and briefly treated with hair removal cream before allowing mice to recover for one day. Prior to inducing wounds, mice were anaesthetized with intraperitoneal injections of 100 µl of a ketamine solution (100 mg/kg) and placed on a water-heated surgical bed. The shaved area was sterilized using alcohol swabs, and 4 equivalent wounds were induced by taking two 6 mm punch biopsies through-and-through the folded dorsal skin. Pictures were taken of the wounds across the course of 10 days; a ruler was included to calibrate the length/pixel ratio. ImageJ software was used for calculating the wound surface area in mm^2^. At the end of the experiment, a second punch biopsy incorporating the wound bed was taken from the euthanized mice and either put in tissue cassettes between sponges and immediately placed in 10% formalin for fixation and subsequent immunohistochemistry procedure or put in 1 ml PBS on ice. Samples in PBS were subsequently homogenized using a Magna-Lyzer instrument (Roche), before plating serial dilutions of the homogenate on THA plates for overnight incubation and enumeration of bacterial CFUs in the wound bed. For the purpose of monitoring FOXO mRNA levels *in vivo*, 6 mm punch biopsies were taken in outbred CD-1 mice as described above. The first biopsies were considered to represent non-wounded skin. At indicated time points, 6 mm punch biopsies were used to acquire the area covering the wound edges and immediately put in 1 ml Trizol on ice and homogenized using a Magna-Lyzer. Samples were then frozen in −80°C until mRNA isolation.

### Lentiviral production

The lentivirus particles used for control and for knock down of FOXO3 transcript was produced according to manufacturers instruction using a lentivirus production kit and the respective control and FOXO3 shRNA constructs with GFP were purchased from the same company (ATCGbio Life Technology).

### Scratch assay with primary human keratinocytes

Primary human keratinocytes were grown in 12 well plates with daily changes of complete KGM medium supplemented with an extra 100 ng/ml recombinant human EGF for 4 days before switching to incomplete KGM medium 2 days before the scratch assay. One day before inducing scratches cells were transduced in 500 µl medium with 1×10^6^ PFU/ml of adenoviral vectors containing either the AAA FOXO3 or the GFP control constructs. Transfection rate was assessed by fluorescence microscopy before inducing scratches and changing medium. For FOXO3 knockdown experiments, lentiviral vectors were used to introduce either an shRNA construct targeting FOXO3 mRNA or a control construct with negative control shRNA. Lentiviral transduction was performed three days before inducing scratches, due to the slower build up of shRNA levels when using the lentiviral approach as compared to the adenoviral transduction. Pictures were taken at indicated time points using a Hamamatsu C4742-95 digital camera mounted on a Nikon Eclipse TE200 microscope with a 4× objective. Pictures were calibrated and analyzed using the NIS Elements D 3.10 imaging software. Cells were lysed using 1 ml Trizol per well and stored in −80°C until mRNA and protein purification was performed.

### Human *in vivo* skin wounds

Punch biopsies of *in vivo* wounded and non-wounded human skin were acquired as previously described [28]. Briefly, after administrating a local anesthesia and informed consent, skin punch biopsies were obtained from healthy donors under protocols approved by the Ethics Committee at Lund University (Lund, Sweden). All procedures in this study involving human samples were performed in accordance with the guidelines in the Declaration of Helsinki Principles. The first biopsy was considered non-wounded skin. *In vivo* skin wound samples were later retrieved after 4 days by performing new punch biopsies covering the edges of the initial biopsies.

### Immunohistochemistry on human and murine *in vivo* wounds

Skin specimens were immediately fixed in 10% formalin for 24 h, dehydrated, and embedded in paraffin. Specimens were sectioned in 4 µm sections using a Leica microtome. Sections were placed on poly-lysine-coated glass slides and placed in an oven at 60°C for 2 h. Slides were then treated with Dako antigen retrieval solution (Dako) for 40 min at 97°C followed by two Hot Rinse washes (Biocare Medical). After blocking in 20% pre-immune serum, the slides were incubated for 24 h at room temperature with a 1∶1000 dilution of either a polyclonal rabbit anti-pFOXO antibody (that recognize FOXO3, FOXO1 and FOXO4 only when phosphorylated on the threonine amino acids at position 32, 24 and 28, respectively LifeSpan Biosciences, Seattle, CA), rat anti-F4/80 (BMA Biomedicals, Switzerland, clone BM8) or rat anti-Gr1 antibodies (R&D Systems, clone RB68-C5). The antibodies were diluted in TBS with 0.05% Tween 20, 1% BSA, and 5% serum from the same species as the secondary antibody. After three 20-minute washes in TBS with 0.05% Tween 20, the slides were incubated with appropriate secondary antibodies diluted 1∶1,000 in the same buffer as the first antibody and incubated for another 24 h followed by three 20-min washes. Secondary antibodies were Alexa 594-conjugated F(ab′)_2_ fragments of goat anti-Rabbit Ig (Life Technologies) and alkaline phosphatase conjugated goat anti-rat antibodies (Jackson Immunoresearch, PA). Slides on human skin wounds were subsequently mounted with Pro long gold with DAPI (Life Technologies) and allowed to cure for 24 h before observation and image acquisition. In case of the murine skin wounds, the slides were first developed for equal time periods using Vulcan Fast Red (Biocare Medical, Concord CA) and thereafter counterstained using Harris hematoxilin (EM Science, Gibbstown, NJ).

### Microscopy

Acquisition of images was performed using a Nikon Eclipse TE200 fluorescence microscope (equipped with a Hamamatsu C4742-95 cooled charge-coupled device camera, using Plan Apochromat 4× 20×, 40× objectives and a 100× objective with N.A 1.4) and a N.A 1.4 oil condenser. The acquisition software used was Nikon NIS-Elements D 3.10. Images were processed using the FIJI open source package [29]. In all figures, acquisition of images was made using the same exposure time for each fluorophore to maintain the relative intensity between non-wounded and wounded skin for each magnification level. During post-processing (only linear changes), the images were treated identically between wounded and non-wounded skin.

### Murine neutrophil killing assay

Dorsal air pouches were created by subcutaneous injection of 2.5 mL sterile air according to an established method [30]. Subsequently, 600 µl of a PBS solution containing 0.5% of carboxymethyl cellulose (sodium salt; Sigma-Aldrich) and 2 µg lipopolysaccharide was injected into the air pouches. After 4 hours, mice were euthanized and the pouches were lavaged with PBS containing 3 mM EDTA. The cells were pelleted at 500×*g* and washed once in PBS before counting them using a hemacytometer. Murine neutrophils were resuspended in RPMI 1640 medium (Invitrogen) containing 2% heat-inactivated fetal calf serum and seeded at 4×10^5^ cells/well. Group A *Streptococcus* (GAS) bacteria grown to logarithmic phase (OD_600_ of 0.4) in THB were diluted to the desired concentration in RPMI 1640 medium+2% heat-inactivated fetal calf serum, then added to the neutrophils at a multiplicity of infection of 1∶10 (GAS- to-neutrophil ratio). Plates were centrifuged at 500×*g* for 10 min and incubated at 37°C in 5% CO_2_ for 30 min or 60 min. The contents of the wells were serially diluted in sterile H_2_O for neutrophil lysis, then plated on THA for enumeration of surviving GAS CFU. Internal control wells without neutrophils were used to determine baseline bacterial counts at the assay endpoints. Percent survival of GAS was calculated as [(CFU/ml experimental well)/(CFU/ml control well)]×100.

### RNA isolation and real-time PCR

Total RNA was isolated and double purified by Trizol (Invitrogen) per manufacturer's recommendations, then precipitated with ethanol and resuspended in 0.1 mM EDTA, with concentration determination by spectrophotometric measurement (Nanodrop 2000). cDNA was synthesized from 200 ng purified RNA using iScript cDNA synthesis kit (Bio-Rad) per manufacturers' instructions. Expression of target genes together with G3PD expression was analyzed using iQ SYBR Green Supermix (Bio-Rad). The primer pairs were all designed to bind at an exon junction. Murine primers were as follows: FOXO1: 5′-ggacagccgcgcaagaccag-3′ and 5′-ttgaattcttccagcccgccga-3′; FOXO3: 5′-gtggaccgacttccgctcgc-3′ and 5′-gcttgccaggatgggcgaca-3′; FOXO4: 5′-actttgagccagatccctgagtcac-3′ and 5′-taaggacaggcctggctccacc-3′; murine GADPH: 5′-gctcggccggctggaagaact-3′ and 5′-ccctcgttctgcacgcggat-3. The human primers were IL-8: 5′-agagacagcagagcacac-3′ and 5′-agttctttagcactccttgg-3′; IL-6: 5′-agaacagatttgagagtag-3′ and 5′-agaatgagatgagttgtc-3′; MMP9: 5′-tgacagcgacaagaagtg-3′ and 5′-cagtgaagcggtacatagg-3′; GADPH: 5′-tggtatcgtggaaggactc-3′ and 5′-agtagaggcagggatgatg-3. Amplification was performed at 58°C for 40 cycles in iCycler Thermal Cycler (Bio-Rad) and data analyzed using iCycler iQ Optical System Software. The relative expression in each sample was calculated by a mathematical method based on the real-time PCR efficiencies [31].

### Statistics

GraphPad Prism 6 was used for statistical analysis in all the validation experiments. Unless otherwise specifically stated, a two-tailed unpaired student t-test was used.

## Results

### TFBS analysis implicates FOXO transcription factors as key regulators during cutaneous wound healing

Since most transcription factors work in conjunction with others, more accurate information regarding gene regulation can be acquired by looking for two to three co-occurring TFBS motifs located closely (e.g. within 50 base pairs) of one another. To verify the feasibility of this approach in the context of wound healing, we completely reanalyzed a published human *in vivo* data set collected during the proliferative phase of cutaneous wound healing [14]. A two-group comparison test was conducted with a cut off q-value of 0.05, resulting in 1,321 remaining probe sets. We reasoned that among the significantly differentially expressed genes in any given data set, the ones giving rise to the highest degree of variance between the studied conditions are likely to be the most important contributors to the particular biological process under study. The likelihood of capturing transcription factors with key roles in the process being studied should therefore increase if one focuses on a selected subset of the significant genes that gives rise to the highest degree of variance in the data set. We therefore chose to study the top fraction of the 1,321 probe set with the highest fold changes in order to reduce noise and with the added benefit that it generated a less computationally intense data analysis. Arbitrarily the top 100 of these up- and down-regulated probe sets giving rise to the highest degree of variance between the non-wounded and wounded skin samples were therefore selected for further analysis, resulting in probe sets with a p-value≤0.008. Of these, 98 were unique annotated genes (**Table S1**). The putative promoter regions (1500 base pairs upstream and 500 base pairs downstream) of these genes were next analyzed with a contemporary version of the now freely available TFBS analysis program SMART [1]. For an overview please see schematic (**Figure 1F**). This analysis revealed a significant enrichment of FOXO1 and FOXO4 TFBS (p<10^−5^), consistent with prior predictions [28]. In addition the current comprehensive analysis revealed an enrichment of FOXO3 TFBSS (p<0.022) (**Table S2**). To test the robustness of these results we also tested increasing the number of probe sets included in the analysis to 150 and 200, which generated similar results (**Table S3 and S4**). We next investigated the presences of co-occuring TFBS for FOXO1, FOXO4 and FOXO3 and detected all three FOXO factors within 50 base pairs of each other in the majority (71%) of the promoter regions of the 98 genes (**Figure 1A, 1B**). Since FOXO transcription factors are known to share binding sites and all bind and recognize the insulin response element (IRE) and the Daf-16 family binding element (DBE) we analyzed the TRANSFAC FOXO TFBS matrixes used in both analysis. We found that they had an identical core motif TGTTT in common but that they all contained dissimilar shores either in length, content or both, reflecting their reported differential preferences for these flanking regions (**Table S5**). As a result quite a few of the co-occurring FOXO1, FOXO3 and FOXO4 TFBS in the genes are partially overlapping as can be seen in **Figure 1A** **and Table S5**. We next corroborated the above findings in an unbiased analysis of the most differentially expressed genes between wounded and non-wounded human epidermis during the *acute* phase of wound healing from another microarray data set published by an independent research group [15](**Table S6, S7**).

Together the highly significant overrepresentation of co-occurring FOXO1, FOXO3 and FOXO4 TFBS in both data sets supported a role for FOXO transcription factors during cutaneous wound healing. We therefore wanted to identify the most suitable FOXO candidate for performing proof-of-principle biological validation experiments with. An ingenuity pathway analysis of FOXO1, FOXO4 and FOXO3 together with the genes identified by the principal component analysis (**Figure 1D**) was performed. All of the co-occurring FOXO transcription factors integrated well in the generated regulatory network proposed for the selected set of genes by the Ingenuity Pathway Analysis software (**Figure 1C**). In particular, FOXO3 has previously been associated with negative regulation of some of the genes occupying key nodes in the generated network, including interleukin-6 (IL-6) [32], [33], IL-8 [34], matrix metalloprotease-1 (MMP-1) [35] and MMP-9 [36], [37]. FOXO3 has also been reported to be able to bind its own promoter and the FOXO1 and FOXO4 promoters, thereby increasing the expression of all three FOXO transcription factors in a positive feedback loop (**Figure 1C**). It has been demonstrated that this loop can be disrupted by platelet-derived growth factor (PDGF) and insulin-like growth factor I (IGF-I) that via downstream SGK/Akt phosphorylation of FOXOs decrease their transcriptional activity [38]. We reasoned that the prolonged EGFR signaling present during wound healing [39]–[43] could in a similar manner inhibit FOXO transcriptional activity. This process would disrupt the positive feedback loop maintained by FOXO3 resulting in a decrease in FOXO3, FOXO1 and FOXO4 mRNA expression levels. We therefore examined the expression levels of FOXO transcription factors during wound healing. Indeed, we detected a decrease in the expression levels of all FOXO transcription factors when comparing the human *in vivo* wounded skin to non-wounded skin (**Figure 1E**); albeit the decrease of FOXO4 did not achieve statistical significance. Notably, the highest baseline expression levels of the FOXO family members prior to wounding were found with FOXO3. We therefore chose FOXO3 as our main validation candidate.

### FOXO transcription factors are phosphorylated to a higher degree and both target genes and known regulators of FOXO3 transcriptional activity are differentially expressed in wounded human skin

**Figure 2:**
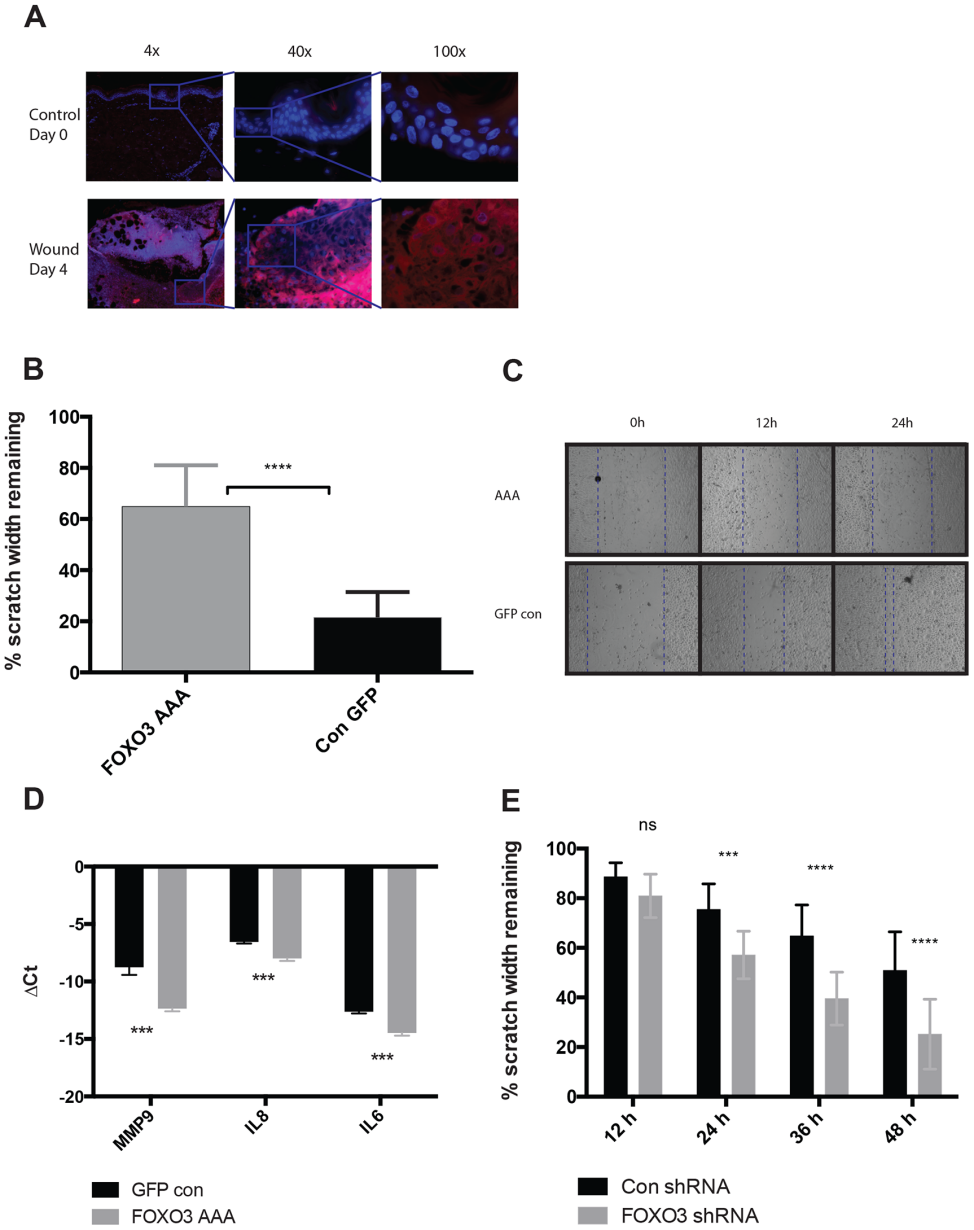
Immunohistochemistry on phosphorylated FOXO transcription factors in wounded human skin and scratched human primary keratinocytes expressing either a constitutively active form of FOXO3 or shRNA targeting FOXO3. A) Immunohistochemistry was performed on *in vivo* wounded human skin four days post wounding as well as on normal skin from the same donor. An anti-pFOXO antibody and a secondary fluorescently labeled antibody was used to detect the levels of phosphorylated FOXO transcription factors in the samples. Notice the increase in fluorescence intensity in the cytoplasm of keratinocytes closest to the front of the migrating epidermal tongue moving in under the wound crust at day 4. B–D) Scratch assays were performed on cultures of primary human keratinocytes transduced with either GFP control or FOXO3 AAA using adenoviral vectors and re-epithelialization was monitored over 24 h. B) Bar graph depicts the percent remaining of the original scratch width after 24 h (n = 9). C) Representative image of scratches from each condition at 0, 12 and 24 h. D) RT-PCR was run on isolated mRNA from scratched wells after 24 h. MMP-9, IL-8 and IL-6 gene expression was normalized using GAPDH (Glyceraldehyde 3-phosphate dehydrogenase) as housekeeping gene. E) Scratch assays were performed on cultures of primary human keratinocytes transduced with either negative shRNA control or shRNA targeting FOXO3 using lentiviral vectors and re-epithelialization was monitored over 48 hours. Bar graph depicts the percent remaining of the original scratch width after 24 h (n = 12).

We next examined the presence of phosphorylated FOXO transcription factors (pFOXO) in *in vivo* wounded human skin using immunohistochemistry. An antibody that recognizes FOXO3, FOXO1 and FOXO4 only in their phosphorylated state (the antibody recognizes phosporylated Thr32, Thr 24 or Thr28 in FOXO3, FOXO1 and FOXO4). We observed an overall increase of pFOXO at the wound site and keratinocytes closest to the migrating edge where more intensely stained (Figure 2A). Having observed an increase in pFOXO in human *in vivo* wounds as well as a significant decrease of FOXO3 in both *in vivo* wounded human and mouse skin, we investigated known, potential and indirect target genes of FOXO3 in the human wound healing proliferative phase data set [14] (**Table 1**). A list of direct target genes whose expression is either repressed or increased by FOXO3 activity have previously been compiled from evidence in various cell types [44]. Paralleling the observed decrease of FOXO3 expression, we found up- or down-regulation of several of these target genes (**Table 1** , bold and marked *) during wounding of human skin *in vivo*. Among FOXO3 target genes involved in regulating cell cycle, we found an increase in FOXM1, microRNA 21 (MIR21) and inhibitor of DNA binding 1 expression and a decrease was observed in cyclin-dependent kinase inhibitor 1B (p27) and retinoblastoma-like 2 expression. We also found a decrease of several FOXO3 target genes known to be involved in autophagy including F-box protein 32 (Atrogin 1), microtubule-associated protein 1 light chain 3beta (MAP1LC3B aka LC3B), GABA(A) receptor-associated protein like 1 (GABARAPL1). It should however be noted that we also found a number of reported target genes of FOXO3 that were also significantly differentially expressed, but did not correlate as predicted with a decrease in FOXO3 activity and/or FOXO3 expression levels (**Table 1** , marked **).

**Table 1:** Significantly differentially expressed target genes of FOXO3.

Gene Symbol	Description	FOXO3 effect	Log2-fold change	*P*-value	Q-value (%)	Pathway	Reference(s)
CCNB1**	cyclin B1	+	2.41	0.025	0	Cell cycle	Alvarez et al. 2001
**CCND1** **	cyclin D1	−	−1.45	0.00002	0	Cell cycle	Schmidt et al. 2002
CCND2*	cyclin D2	−	1.02	0.001	0	Cell cycle	Fernandez de Mattos et al. 2004
CCND3*	cyclin D3	−	0.54	0.01	0.7	Cell cycle	Kornblau et al. 2010
**RBL2** *	retinoblastoma-like 2 (p130)	+	−0.88	0.0051	0.5	Cell cycle	Chen et al. 2006; Kops, et al. 2002
**CDKN2D** **	cyclin-dependent kinase inhibitor 2D (p19, inhibits CDK4)	+	0.42	0.018	1.3	Cell cycle	Katayama et al. 2008
**CDKN1B** *	cyclin-dependent kinase inhibitor 1B (p27, Kip1)	+	−0.7	0.01	0.9	Cell cycle	Dijkers, et al. 2000
**CAT** *	catalase	+	−0.44	0.04	2.5	Stress resistance	Nemoto & Finkel 2002
**PRDX3** **	peroxiredoxin 3	+	0.76	0.006	0.2	Stress resistance	Chiribau et al. 2008
**GADD45A** **	growth arrest and DNA-damage-inducible, alpha	+	0.55	0.02	1.1	DNA repair	Tran et al. 2002, Furukawa-Hibi et al. 2002
**BCL2L11** **	BCL2-like 11 (BIM, apoptosis facilitator)	+	0.39	0.008	1.2	Apoptosis	Dijkers, et al. 2000; Gilley et al. 2003
**TNFSF10** **	tumor necrosis factor ligand superfamily, member 10 (TRAIL)	+	0.84	0.00074	0	Apoptosis	Modur et al. 2002
**G6PC3** **	glucose 6 phosphatase, catalytic, 3	+	0.45	0.00013	0.3	Metabolism	Onuma et al. 2006
**ID1** *	inhibitor of DNA binding 1	−	1.5	0.05	0.6	Differentiation	Birkenkamp et al. 2007
**FBXO32** *	F-box protein 32 (Atrogin 1)	+	−0.72	0.034	0.8	Autophagy	Sandri et al. 2004
**BNIP3** **	BCL2/adenovirus E1B 19 kD interacting protein 3	+	1.13	0.002	0.1	Autophagy	Mammucari et al. 2007; Zhao et al. 2007
**MAP1LC3B** *	microtubule-associated protein 1 light chain 3 beta	+	−0.37	0.0064	1.1	Autophagy	Mammucari et al. 2007; Zhao et al. 2007
**GABARAPL1** *	GABA(A) receptor-associated protein like 1	+	−0.83	0.0035	0.2	Autophagy	Zhao et al. 2007
ULK2*	unc-51-like kinase 2	+	−0.44	0.0011	0.5	Autophagy	Zhao et al. 2007
ATG14*	ATG14 autophagy related 14 homolog	+	−0.56	0.007	0.6	Autophagy	Xiong et al. 2012
**EIF4EBP1** **	eukaryotic translation initiation factor 4E binding protein 1	+	1.69	0.0044	0	Insulin signaling	Puig et al. 2003
**PIK3CA** *	phosphoinositide-3-kinase, catalytic, alpha polypeptide (p110a)	+	−1.34	0.0002	0	Signaling	Hui et al. 2008
IL6*	interleukin 6	−	4.51	0.017	0	Inflammation, wound healing	Dejean et al. 2009; L. Lin et al. 2004
**MMP1** **	matrix metallopeptidase 1 (interstitial collagenase)	+	11.98	0.041	0	Wound healing	Mawal-Dewan et al. 2002
**MMP9** **	matrix metallopeptidase 9 (gelatinase B)	+	3.4	0.02	0	Wound healing	Storz et al. 2009
**MIR21** *	microRNA 21	−	3.23	0.003	0	Wound healing, cell Cell cycle	Wang & Li 2010; Wang et al. 2011
**MXI1** *	MAX interactor 1	+	−0.59	0.0044	0.7	Tumor Supression	Delpuech et al. 2007
**FOXO1** *	forkhead box O1A (rhabdomyosarcoma)	+	−0.86	0.003	0.2	Tumor Supression	Essaghir et al. 2009)
**FOXO3** *	forkhead box O3A	+	−0.64	0.00071	0.3	Tumor Supression	Essaghir et al. 2009)
**CITED2** *	Cbp/p300-interacting transactivator	+	−0.81	0.013	0.5	Angiogenesis	Bakker et al. 2007
**FOXM1** *	forkhead box M1	−	1.65	0.0044	0	stem/progenitor cell expansion	McGovern et al. 2009

List of significantly differentially expressed known, potential and indirect target genes of FOXO3 in Roupé et al 2009 data set comparing human *in vivo* wounded skin with control. Genes known to be direct target genes of FOXO3 are **in bold**.

*Change in expression is consistent with a decrease in FOXO3 transcriptional activity.

**Change in expression not consistent with a decrease in FOXO3 transcriptional activity.

We next investigated the expression of genes coding for signaling pathway molecules and proteins known to regulate the transcriptional activity of FOXOs. Several of these genes were found to be differentially expressed, and notably we identified a marked increase in the expression of AKT1 and SGK1, the two growth factor signaling pathway molecules that once activated, phosphorylate FOXOs, decreasing the affinity to their TFBS and enabling interaction with 14-3-3 proteins. Several of the 14-3-3 proteins mediating nuclear export of FOXO transcription factors also had significantly increased mRNA levels in wounded skin, as did 3-phosphoinositide dependent protein kinase-1 (PDPK1) and members of the mTORC2 complexes needed for AKT1 and SGK1 activation. Conversely mRNA expression levels for the genes encoding serine/threonine-protein phosphatase 2A members that can dephosphorylate FOXO transcription factors were down-regulated along with sirtuin 1 (**Table 2**). The triggering of growth factor signaling pathways and subsequent AKT/SGK activation is known to be the major cue for decreasing the transcriptional activity of FOXOs. The observed changes in the mRNA levels of AKT1 and SGK1 could therefore impart another level of FOXO regulation acting through an altered sensitivity to growth factor stimulus.

**Table 2:** Significantly differentially expressed regulators of FOXO3.

Gene symbol	Description	Mode of action	Effect on FOXO3a activity	Log2-fold change	*P*-value	Q-value (%)	References
***Transcriptional Regulators***							
STAT3**	signal transducer and activator of transcription 3	Transcription	mRNA level+	1.22	0.006	0	Zhao et al. 2007; Oh et al. 2011
FOXO3*	forkhead box O3A	Transcription	mRNA level +	−0.64	0.00071	0.25	Essaghir et al. 2009
***Nuclear Export by FOXO3 Phosphorylation***							
PDPK1*	3-phosphoinositide dependent protein kinase-1	Activates AKT and SGK	Nuclear localization −	0.46	0.03	1.72	Park et al. 2009
***mTORC2 Complex Proteins***							
MLST8*	MTOR associated protein, LST8 homolog	Complex activates AKT and SGK	Nuclear localization −	0.95	0.025	0.51	Guertin et al. 2006; Facchinetti et al. 2008
MAPKAP1*	mitogen-activated protein kinase associated protein 1 (mSIN1)	Complex activates AKT and SGK	Nuclear localization −	0.43	0.0005	0.42	N/A
AKT1*	v-akt murine thymoma viral oncogene homolog 1(PKB)	Phosphorylation	Nuclear localization −	0.75	0.024	0.7	Biggs et al. 1999
SGK1*	serum/glucocorticoid regulated kinase	Phosphorylation	Nuclear localization −	2.01	0.0039	0	Brunet et al. 2001
SGK3**	serum/glucocorticoid regulated kinase family, member 3	Phosphorylation	Nuclear localization −	−0.99	0.012	0.5	McCormick et al. 2004
MAP2K1*	mitogen-activated protein kinase kinase 1	Activates ERKs	Nuclear localization −	0.4	0.018	1.4	Yang et al. 2008
***Promoting Nuclear Localization by Phosphorylation/De-Phosphorylation***							
STK4**	serine/threonine kinase 4 (MST1)	Phosphorylation	Nuclear localization +	0.57	0.044	2.8	Lehtinen et al. 2006
MAPK14**	mitogen-activated protein kinase 14 (p38α)	Phosphorylation	Nuclear localization +	0.59	0.04	1.37	Cai et al. 2008
PPP2CA*	protein phosphatase 2, catalytic subunit, α isozyme	De-phosphorylation	Nuclear localization +	−0.65	0.021	0.7	Singh et al. 2010
PPP2R2A*	protein phosphatase 2, regulatory subunit B, α	De-phosphorylation	Nuclear localization +	−0.56	0.006	0.5	Singh et al. 2010
PPP2R2B*	protein phosphatase 2, regulatory subunit B, β	De-phosphorylation	Nuclear localization +	−1.13	0.004	0.3	Singh et al. 2010
***Acetylation***							
SIRT1*	Sirtuin-1	De-acetylation	Transcriptional activity+	−0.67	0.024	1.2	Brunet et al. 2004
***14-3-3 proteins***							
YWHAG*	tyrosine 3-monooxygenase/tryptophan 5-monooxygenase activation protein, gamma	Binding	Nuclear export +	0.49	0.017	1.1	Dobson et al. 2011
YWHAH*	tyrosine 3-monooxygenase/tryptophan 5-monooxygenase activation protein, eta	Binding	Nuclear export +	0.6	0.008	0.6	Dobson et al. 2011
YWHAQ*	tyrosine 3-monooxygenase/tryptophan 5-monooxygenase activation protein, theta	Binding	Nuclear export +	0.48	0.0003	0.2	Dobson et al. 2011
YWHAZ*	tyrosine 3-monooxygenase/tryptophan 5-monooxygenase activation protein, zeta	Binding	Nuclear export +	0.52	0.02	0.9	Dobson et al. 2011
SETD7*	SET domain containing lysine methyltransferase 7	Methylation	Transriptional activity+	−0.31	0.006	1.4	Calnan et al. 2011

List of significantly differentially expressed genes involved in regulation of FOXO3 expression/activity in the Roupé et al 2009 data set comparing human *in vivo* wounded skin with control.

*Change in expression is consistent with a decrease in FOXO3 activity/expression.

** Change in expression is not consistent with a decrease in FOXO3 activity/expression.

### A constitutively active form of FOXO3 has a negative effect on scratch closure in human primary keratinocytes whereas knockdown of FOXO3 has the opposite effect

To investigate whether FOXO transcription factors could be important for regulation of cutaneous wound healing, we cultured human primary keratinocytes and transduced them 1 day before confluency with an adenoviral vector containing GFP and the constitutively active form of FOXO3 (AAA), in which its three phosphorylation sites (Thr32, Ser253 and Ser315) have been substituted for alanine residues to mimic a non-phosphorylated status [45]. An empty vector containing only GFP was used as a negative control. Upon performance of a scratch assay on the confluent keratinocyte monolayers, we observed a significant decrease in the scratch closure rate in the keratinocytes transduced with FOXO3 AAA compared to the GFP control after 24 h (**Figure 2B, 2C**). Furthermore, we investigated the expression levels of the genes encoding IL-8, IL-6 and MMP-9, all known to both be important for cutaneous wound healing, and observed in our bioinformatics analysis to potentially be suppressed by FOXO3 in keratinocytes. We found a significant decrease in the expression of all three genes in the scratched keratinocytes transduced with FOXO3 AAA compared to control (**Figure 2D**). We next investigated what effects a knockdown of FOXO3 could have on scratch closure using a lentiviral vector that introduced a shRNA construct targeting FOXO3 mRNA along with GFP to estimate the transduction rate. We observed a significant increase in the scratch closure rate in the wells where FOXO3 was knocked down as compared to the wells transduced with scrambled shRNA control virus (**Figure 2E, 2F**). Over 48 hours we observed a mean difference of 25% in closure rate.

### Expression levels of FOXO transcription factors drop in *in vivo* wounded mouse skin

**Figure 3:**
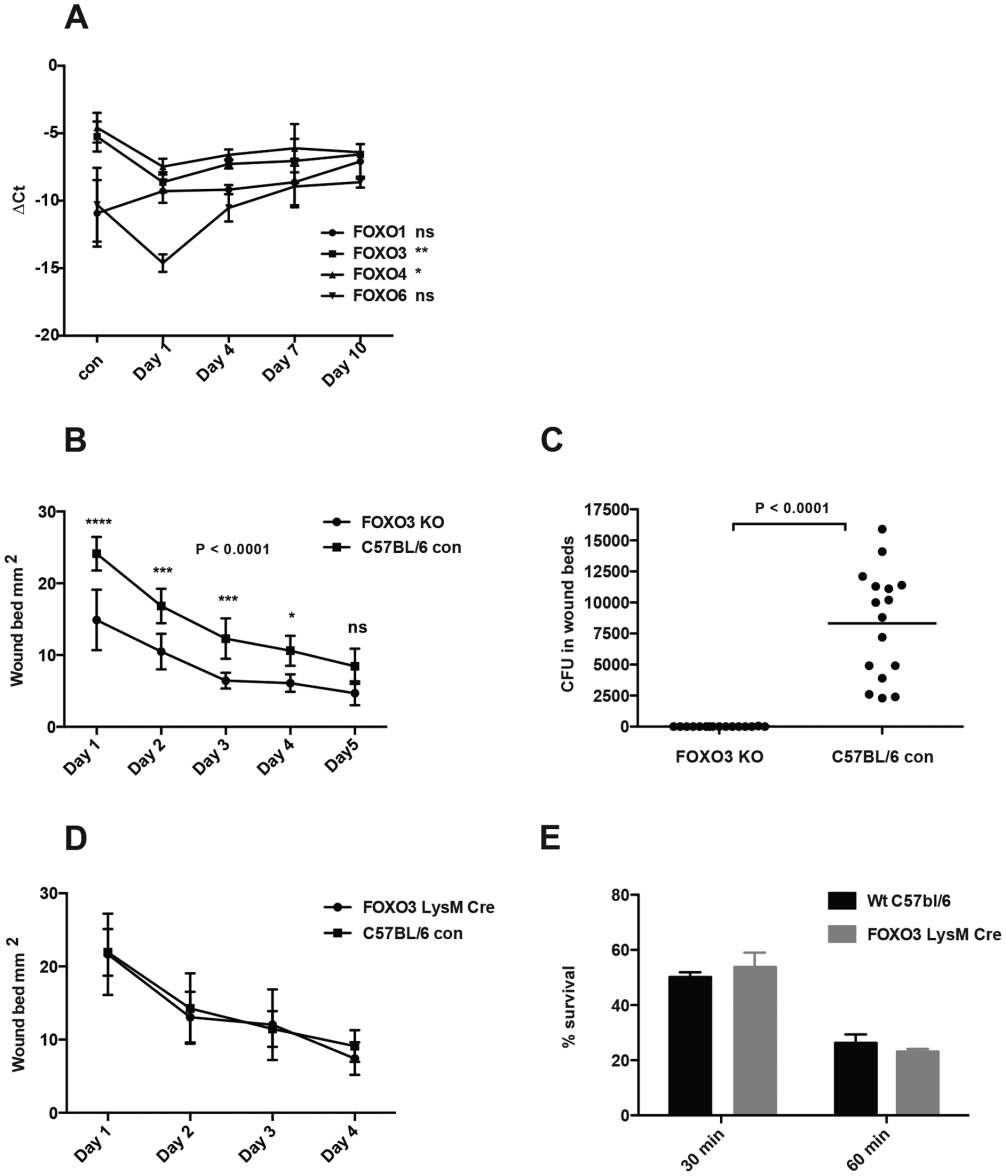
Wound healing rate in FOXO3 and FOXO3 LysM-cre knockout mice compared to control. A) Quantitative RT-PCR using mRNA from non-wounded and *in vivo* wounded mouse skin was performed. All data was normalized to GAPDH expression (Glyceraldehyde 3-phosphate dehydrogenase) that was used as housekeeping gene. A one-way analysis of variance was performed. Significant differences were found day 1 after wounding for FOXO3 and FOXO4 when performing a Dunnett's Multiple Comparison Test against non-wounded control. (*P<0.05, **P<0.01). Error bar denotes mean ±SD (n = 3). B) Graph displays wound size in mm^2^ over time. Values from each mouse represent an average of 4 wounds induced by 6 mm punches through folded dorsal skin. A two-way ANOVA was performed to detect differences over time between FOXO3 knock out mice and C57bl/6 control mice using Bonferroni post-testing to detect differences at each time point. An overall difference was detected over time *P*<0.023 and post-testing generated significant results for day 1 to day 4. (*P<0.05, **P<0.01, ***P<0.001) (n = 4) C) Bacterial loads in the wound beds day 10 after wounding. D) Wound healing rate in FOXO3 LysM-cre mice and control mice over time. E) Group A streptococcal (GAS) survival in neutrophil killing assays using neutrophils isolated from either FOXO3 LysM-cre mice or C57bl/6 control mice. Percent survival is expressed relative to GAS survival with no neutrophils present.

Since we had observed a decrease in FOXO transcription factor expression in wounded human skin *in vivo* at day 4, we monitored FOXO transcription factor expression in wounded mouse skin *in vivo* over a 10-day period. Concordant with the human data, there was a significant drop in FOXO3 and FOXO4 transcription factors expression levels in mouse skin in response to wounding (**Figure 3A**), whereas FOXO1 showed a non-significant increase (**Figure 3A**). The relative expression levels of FOXO4 were found to be higher at baseline when compared to human skin, but the most significant drop in expression levels once again could be seen in FOXO3 levels at day 1, with a 10-fold down-regulation of transcript compared to the unwounded control, p<0.02 (**Figure 3A**).

### The wound healing rate in mice lacking FOXO3 is accelerated

To investigate what role the decrease in FOXO transcription factor expression could play during wound healing *in vivo*, we performed wounding experiments in FOXO3 knockout mice that are known to be viable and grossly indistinguishable from their littermate controls [25]. Specifically, we induced 6 mm punch wounds on the shaved back of the mice, and photographed the wounds over time, to calculate wound area based upon photo calibration and ruler measure. In this study, we observed a significantly accelerated rate of wound healing in mice lacking FOXO3 (p<0.001) compared to wild-type littermate controls (**Figure 3B**). Furthermore, as the wound experiments were performed under routine husbandry conditions in a non-sterile environment, we assessed the bacterial loads in the wound beds 10 days after wounding, observing a clear reduction of bacterial loads (predominantly Gram-positive species) in the wound beds of the FOXO3 −/− mice (**Figure 3C**).

### The wound healing phenotype in FOXO3 knock out mice is not due to differences in macrophage and neutrophil function

Although FOXO3-deficient mice are grossly indistinguishable from their littermate controls, they have been observed to have elevated numbers of granulocytes and macrophages [32] and to develop neutrophilia with age or in response to myeloid stress [46]. Since neutrophils and macrophages have been reported to be involved in wound healing, we next examined the wound healing rate in FOXO3 LysM-cre mice, which harbor a conditional knockout of FOXO3 linked to the expression of the LysM-gene, resulting in high deletion efficiency in granulocytes (e.g. neutrophils) and macrophages [27]. We could not detect any significant difference in the rate of wound healing rate between these groups of mice (**Figure 3D**). Furthermore, we did not detect a difference in the ability of FOXO3 LysM-cre neutrophils to kill a Gram-positive bacterial pathogen (Group A *Streptococcus*) (**Figure 3E**). We also performed immunohistochemistry on the punch biopsies from FOXO3 knockout mice and control mice with a marker against monocytes/macrophages (F4/80) and a marker mainly for neutrophils (Gr1) (**Figure S1A and S1B**). No discernable difference in infiltrating inflammatory cells could be seen at the wound site at day 4 nor could a difference in neutrophil and macrophage numbers be seen in non-wounded skin. Together these results suggest that the accelerated wound healing phenotype in the global FOXO3 knockout mice is likely not due to altered functions of neutrophils and macrophages, but rather to processes in endogenous cutaneous cells such as keratinocytes.

## Discussion

We set out to investigate whether a search for enriched TFBS and the presence of clustered transcription factor binding sites in the promoter regions of the most differentially expressed genes in data sets could identify novel key regulators of complex biological process. Using this approach we identified FOXO transcription factors as potential key regulators of the epidermis during the wound healing process, and showed that mice lacking FOXO3 exhibit a significantly accelerated wound healing rate. FOXO3 has to the best of our knowledge not previously been reported to play a role in wound healing.

Though speculative, our finding that the absence of FOXO3 is beneficial for the wound healing process in mice *in vivo* could possibly contribute to explaining some of the pathophysiology of impaired wound healing seen in the elderly. Increased FOXO3 nuclear localization is positively correlated with age [47], [48]. One could therefore envision that this shift towards more nuclear FOXO3 could increase the threshold of HB-EGF shedding and other growth factors needed to release the FOXO3 break on keratinocyte proliferation, leading to a decreased ability to proficiently respond to cutaneous injuries. Impaired wound healing can further be complicated with the onset of age-related type 2 diabetes [49]. Insulin receptor signaling has been found to be nearly absent in non-wounded and wounded skin of diabetic mice and this has also been shown to severely impede wound healing [50]. Since FOXO activity is negatively regulated by the insulin receptor through AKT activation [51] one could similarly speculate whether this could contribute to a baseline of FOXO activity in diabetes that is less sensitive to the growth factor stimuli necessary for efficient wound healing. Concordantly it has been reported that TNF-alpha signaling is associated with decreased wound healing rates in diabetic mice and accompanied by high nuclear levels of FOXO1 in fibroblasts [52]. Topical treatment with insulin has also recently been shown to enhance AKT and ERK pathways in rats and to accelerate wound closure in diabetic patients in a double-blind placebo-controlled clinical trial [53]. If one disregards treatments based on antibiotic regimens or wound dressings, then PDGF (Regranex) has for a long time been the only approved drug currently on the market for promoting wound healing in diabetic foot ulcers; however, this drug comes with a black box warning. Encouragingly, late stage clinical trials for cutaneous application of other growth factors also present during wound healing e.g. fibroblast growth factor (FGF) (CVBT-141 CardioVascular BioTherapeutics), basic fibroblast growth factor (bFGF) (Trafermin) and EGF (Daewoong pharmaceuticals), VEGF Telbermin are under way. These growth factors have in common that they can target keratinocytes, as opposed to PDGF [54], and that they, like insulin and PDGF, all increase Akt signaling which in turn will decrease FOXO activity and FOXO expression [38]. The receptors for these growth factors have all been targeted for anticancer therapies. However only mild decreases in wound healing rates [55], [56] have so far been reported for antagonists of these receptors, which could be due to a lack of studies specifically focused on wound healing or due to a functional redundancy of growth factors present at the wound site.

The sustained growth factor and EGFR signaling known to be present during normal wound healing [39]–[43] is liable to cause a mainly cytosolic localization of FOXO transcription factors through the downstream activation of AKT and SGK. Interestingly we found up-regulated expression of PDK1, members of the mTORC2 complex, 14-3-3 proteins and importantly AKT1 and SGK1 in *in vivo* wounded human epidermis as well as other regulators of FOXO activity (**Table 2**). These changes could during normal wound healing contribute to further negatively regulate FOXO transcriptional activity by increasing keratinocytes responsiveness to the abundant growth factor signaling present during the wound healing process [39]–[43].

The overall decrease of FOXO transcription factor expression levels in response to wound healing we observed *in vivo* is consistent with previous mechanistic findings that have shown that FOXO3 exerts a positive feedback loop on its own expression as well as the expression of FOXO1 and FOXO4 [38]. FOXO1 is in turn known to positively regulate its own expression which could further amplify the decrease of FOXO3 on FOXO1 expression [57]. FOXO3 had the highest expression level in non-wounded human skin (**Figure 1E**). The prolonged growth factor and EGFR signaling present during wound healing [39]–[43] and the subsequent inactivation of FOXO3 activity by AKT/SGK phosphorylation and nuclear export, is therefore likely one of the major reasons underlying the observed decreases in expression levels of FOXO1, FOXO3 and FOXO4 transcription factors. This finding aligns with the increase in pFOXO we observe at the wound site (**Figure 3B**).

The differential expression of genes known to be induced or suppressed directly by FOXO3 e.g. ID1 [58], RBL2 [59], [60], CDKN1B [61], CAT [62], FBOX32[63], MAP1LC3B [64], [65], FOXM1[66] and MIRN21 [67] is consistent with a decrease in FOXO3 activity and expression. Interestingly MIRN21 is known to increase re-epithelialization and keratinocyte migration [68] and is like FOXO3 involved in regulation of apoptosis and the cell cycle at multiple levels [69]. Counter-indicative of a predominantly cytosolic localization of FOXO3 during cutaneous wound healing is the increased expression of MMP1, MMP9 and other target genes that have been found to be positively regulated by FOXO3 [70], [71]. However for e.g. MMP1 and MMP9 recent reports have associated a decrease of FOXO3 activity/expression with an increase in MMP1 and MMP9 expression in prostate cancer and in skin in response to UV-damage [35]–[37] which could be illustrating some of the limitations in trying to extrapolate specific roles for FOXO transcription factors between different tissues and cell types [20].

The human cutaneous wound healing process is difficult to study due to the interplay of several cell types and the timed expression of several growth factors that are not easily simulated *in vitro*. Several prior gene expression studies on wounded skin [8]–[13] have analyzed *in vitro* cultures or full thickness wounds, making it difficult to delineate pathways from signals originating from individual cell types. Therefore the two data sets we used for analysis were from samples were care had been taken to specifically isolate the epidermis, thereby generating a keratinocyte specific signal [14], [15]. However, even with the most well-defined data sets such as these, an inherent limitation of gene expression profiling is that it does not capture posttranslational events, which regulate the activity of many transcription factors. Another weakness common to the standard analysis procedure of microarrays is the establishment of stringent cutoffs, set to generate manageable data volumes. Consequently, transcription factors are often underrepresented in the published analyses, since changes in the expression of these genes are often minor, yet can have a high impact on multiple levels. These two limitations may partly be the reason why FOXO3 has not previously been implicated in the cutaneous wound healing process, and the finding illustrates the major advantages of our approach for finding novel key regulators.

Since re-epithelialization is dependent on cell proliferation, it is perhaps intuitive that a decreased activity of a tumor suppressor involved in cell cycle arrest is beneficial for wound healing. However in the list of the most differentially expressed genes between wounded and non-wounded epidermis having a significant enrichment of FOXO transcription factor motifs, only a limited number of genes were found to be involved in cell cycle regulation. Instead, the list contained up-regulation of several genes known to be involved in innate immunity, and the epidermal layer of keratinocytes represents a first defense against the surrounding microbial world. These gene included e.g. the antimicrobial peptides human beta defensin 2 (DEFB4), Psoriasin (S100A7), calgranulin A and B (S100A8,S100A9) and Koebnerisin (S100A7A) as well as IL-20 and IL-24 and the pro-inflammatory cytokines IL-6 and IL-8 [72], [73] (**Table S1 and** **Figure 1A**) all described in the original publications of the data set [14], [28]. A rapid response with heightened immune surveillance in response to injury is important to prevent secondary wound infection. While we saw no frank signs of uncontrolled infection during the wounding experiments, we observed a decrease in abundance of colonizing bacteria in the healing wound on day 10 in the mice lacking FOXO3 compared to wild-type control. This difference was not observed in myeloid-cell specific FOXO3 knockout mice, and FOXO3 did not affect neutrophil bacterial killing. Thus the difference in bacterial loads in the wound bed can likely be attributed to keratinocyte specific effects and a faster closure of the wound. An increased ability of keratinocytes in FOXO3 knock out mice to produce antimicrobial peptides and to recruit neutrophils through a more rapid and heightened production of IL-8 [14] and IL-6 [74] in response to wounding could also be a contributing factor.

The decreased re-epithelialization rate in the human primary keratinocyte cultures transduced with the constitutively active form of FOXO3 points towards an important contribution of keratinocytes in the observed wound healing phenotype in FOXO3 knockout mice. Increased proliferation of dermal fibroblasts and/or an increased neovascularization due to the role of FOXO3 in vascularization [74] may be other factors contributing to this phenotype. Future studies in conditional FOXO3 knockout mice targeting keratinocytes, fibroblasts and endothelial cells could help elucidate the respective roles of FOXO3 in these cell types during wound healing. The advent of drugs effecting FOXO transcription factor activity could in light of this study be contemplated to be relevant in the wound healing setting; for example, a low molecular compound inhibiting the transactivation domain of FOXO1 has recently been reported [75].

In summary, searching for significantly over-represented TFBS and co-occurring TFBS in the promoters of the most differentially expressed genes in a data set can as shown in this paper be instrumental in finding important novel regulators of complex biological process. With the accumulating wealth of data daily published in public databases this approach can hopefully contribute to advancing our understanding of key regulatory factors in complex biological process during both disease and development.

## Supporting Information

Figure S1 **Immunohistochemistry on samples of non-wounded and** ***in vivo*** **- wounded murine skin from FOXO3 knockout and wild-type mice.** Samples of non-wounded and *in viv* –wounded murine skin from FOXO3 knockout and wild-type mice were immunostained for markers of macrophages and neutrophils. Non-wounded skin was obtained by punch biopsy. New biopsies of the wound samples were taken on day 4 around the edges of the initial biopsy. Color was developed with Vulcan Fast Red Chromogen and Harris Hematoxylin was used for counterstaining. A) Immunohistochemistry was performed on non-wounded murine skin and in vivo wounded skin 4 days post wounding using antibodies against F4/80, a marker for monocytes and macrophages B) Immunohistochemistry using antibodies against Gr1, a marker for murine neutrophils.
(TIF)

Table S1 **List of the 100 most differentially expressed genes between** ***in vivo*** **wounded and non-wounded human skin at day 4 in the E-MEXP-3305 data set.** For selection criteria please see the methods section for further details.
(DOCX)

Table S2 **List of the 100 most enriched transcription factor binding sites in the promoter regions of the 100 most differentially expressed genes between wounded and non-wounded skin during the proliferative phase of wound healing.** The list of 100 genes used for SMART analysis (see **Table S1**) was acquired by a complete re-analysis of the data set previously published by Roupé et al 2009 (see Materials and Methods). FOXO transcription factors have been highlighted in yellow.
(DOCX)

Table S3 **List of the 100 most enriched transcription factor binding sites in the promoter regions of the 150 most differentially expressed genes between wounded and non-wounded skin during the proliferative phase of wound healing.** FOXO transcription factors have been highlighted in yellow. Please see the methods section for further details.
(XLSX)

Table S4 **List of the 100 most significantly enriched transcription factor binding sites in the promoter regions of the 200 most differentially expressed genes between wounded and non-wounded skin during the proliferative phase of wound healing.** FOXO transcription factors have been highlighted in yellow. Please see the methods section for further details.
(XLSX)

Table S5 **The genes among the most differentially expressed genes between wounded and non-wounded skin that contain enriched co-occurring transcription factor binding sites of FOXO1, FOXO3 and FOXO4 sites in their promoter regions.** List of the genes from Table S1 which were found to contain within 50 base pairs of each other. The list also contains information were these binding sites were found (position 1 denotes 1500 upstream and 2000 is 500 base pairs down stream with the opposite for strand 2). In addition clusters containing only two of either one of the three FOXO transcriptions factor binding sites within 50 basepairs of each other has been included. The binding motif for each site are also listed as are the core and matrix sequence scores.
(XLSX)

Table S6 **The 100 most differentially expressed genes between wounded and non-wounded skin during the acute phase of wound healing adapted from the list published by Kennedy-Crispin et al 2011.** Since the list from Kennedy-Crispin et al 2011 originally contained several duplicates and 2 genes also were excluded from analysis the resulting list used for analysis contained the 88 genes in the table. Genes with increased expression are colored red and genes with decreased expression are colored blue. The 46 genes containing co-occurring FOXO1, FOXO4 and FOXO3 transcription factor binding sites are marked with *.
(DOCX)

Table S7 **List of the 100 most enriched transcription factor binding sites in the promoter regions of the most differentially expressed genes between wounded and non-wounded epidermis published by Kennedy-Crispin et al 2011.** FOXO transcription factors have been highlighted in yellow.
(DOCX)
