# Supplementary figures and images for: Transcription Factor Binding Site Analysis Identifies FOXO Transcription Factors as Regulators of the Cutaneous Wound Healing Process
Source: PLoS One. 2014 Feb 19;9(2):e89274. doi: 10.1371/journal.pone.0089274 (PMC3929751; doi:10.1371/journal.pone.0089274)

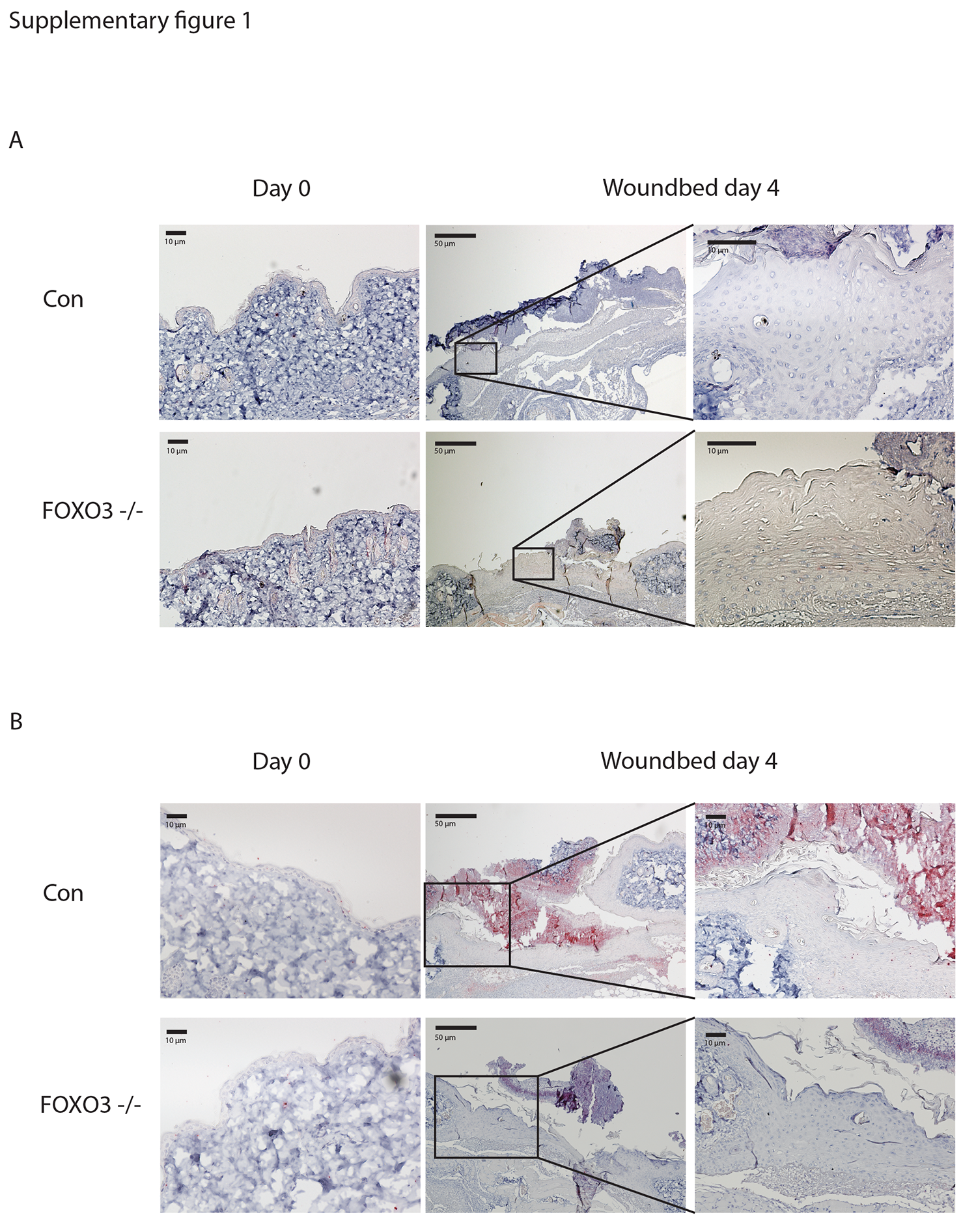

Supplement: Figure S1 — Immunohistochemistry on samples of non-wounded and in vivo - wounded murine skin from FOXO3 knockout and wild-type mice. Samples of non-wounded and in viv –wounded murine skin from FOXO3 knockout and wild-type mice were immunostained for markers of macrophages and neutrophils. Non-wounded skin was obtained by punch biopsy. New biopsies of the wound samples were taken on day 4 around the edges of the initial biopsy. Color was developed with Vulcan Fast Red Chromogen and Harris Hematoxylin was used for counterstaining. A) Immunohistochemistry was performed on non-wounded murine skin and in vivo wounded skin 4 days post wounding using antibodies against F4/80, a marker for monocytes and macrophages B) Immunohistochemistry using antibodies against Gr1, a marker for murine neutrophils. (TIF) [file pone.0089274.s001.tif]
