# Supplementary material for: Transcription Factor Binding Site Analysis Identifies FOXO Transcription Factors as Regulators of the Cutaneous Wound Healing Process
Source: PLoS One. 2014 Feb 19;9(2):e89274. doi: 10.1371/journal.pone.0089274 (PMC3929751; doi:10.1371/journal.pone.0089274)
Supplement: Table S1 — List of the 100 most differentially expressed genes between in vivo wounded and non-wounded human skin at day 4 in the E-MEXP-3305 data set. For selection criteria please see the methods section for further details. (DOCX) [file pone.0089274.s002.docx]

**Supplementary Table 1**

List of the 100 most differentially expressed genes between *in vivo* wounded and non-wounded human skin at day 4 in the E‑MEXP‑3305 data set. Please see the methods section for further details on selection criteria.

| **Affymetrix Probe I.D.** | **Gene Symbol** | **Description** | **Log2-fold change** | **Q-value (%)** | **Definition** | **Function** |
| --- | --- | --- | --- | --- | --- | --- |
| 41469_at | MMP1 | matrix metallopeptidase 1 | 9.9 | 0 | Matrix metalloprotease | Keratinocyte migration |
| 232170_at | DEFB4A | defensin, beta 4 | 9.8 | 0 | Antimicrobial peptide | Antimicrobial activity, Induced in wounded skin |
| 207356_at | SPP1 | secreted phosphoprotein 1 (osteopontin) | 8.2 | 0 | Cytokine | Cytokine that upregulates expression of interferon-gamma and interleukin-12 |
| 211906_s_at | S100A7A | S100 calcium binding protein A7-like 1 | 7.7 | 0 | S100 protein | Involved in epidermal differentiation |
| 209720_s_at | SERPINB4 | serpin peptidase inhibitor, clade B, member 4 | 7.2 | 0 | Peptidase inhibitor | Inhibits granzyme M-induced cell death |
| 204351_at | PI3 | peptidase inhibitor 3, skin-derived (SKALP) | 7.2 | 0 | Peptidase inhibitor, antimicrobial | Elastase-specific inhibitor antimicrobial peptide against Gram+ and Gram- bacteria |
| 232082_x_at | IL8 | interleukin 8 | 6.5 | 1.4E-01 | Interleukin | Attracts neutrophils to the wound bed |
| 209800_at | S100A9 | S100 calcium binding protein A9 (calgranulin B) | 6.3 | 0 | S100 protein, Antimicrobial | Antimicrobial activity towards bacteria and fungi |
| 206561_s_at | IL24 | interleukin 24 | 5.9 | 0 | Interleukin | Terminal cell differentiation, overexpression leads to elevated expression of GADD family genes |
| 223278_at | TCN1 | transcobalamin I (vitamin B12 binding protein) | 5.8 | 5.6E-02 | Antimicrobial peptide | Antimicrobial activity, facilitates the transport of cobalamin into cells |
| 209773_s_at | IL20 | interleukin 20 | 5.7 | 0 | Interleukin | Cytokine that may be involved in epidermal function and psoriasis. Acts through STAT3 |
| 224910_at | SPRR3 | small proline-rich protein 3 | 5.3 | 0 | Small proline rich protein | Cross-linked envelope protein of keratinocytes, important for barrier formation |
| 208539_x_at | KRT16 | keratin 16 | 5.0 | 0 | Cytokeratin | Paired with keratin 6, forms intracellular intermediate filaments |
| 204268_at | GZMB | granzyme B | 5.0 | 0 | Enzyme | Proapoptotic, target cell lysis in cell-mediated immune responses, cleaves caspase-3, -7, -9 and 10 |
| 206172_at | MMP10 | matrix metallopeptidase 10 | 4.8 | 0 | Matrix metalloprotease | Involved in keratinocyte migration |
| 209875_s_at | PRSS27 | protease, serine 27 | 4.8 | 0 | Serine protease | Tryptic serine peptidase that cleaves peptides after an arginine residue |
| 235075_at | IGFL1 | IGF-like family member 1 | 4.6 | 0 | Growth factor | Induced during inflammatory skin conditions |
| 226817_at | SERPINE1 | serpin peptidase inhibitor, clade E, member 1 | 4.6 | 0 | Peptidase inhibitor | Inhibits plasminogen activator, urokinase, protein C and matriptase-3/TMPRSS7 |
| 232074_at | SERPINB3 | serpin peptidase inhibitor, clade B, member 3 | 4.5 | 0 | Peptidase inhibitor | May act as a protease inhibitor to modulate the host immune response |
| 203535_at | IL6 | interleukin 6 | 4.5 | 0 | Interleukin | Produced at sites of acute and chronic inflammation, Cytokine with a wide variety of biological functions |
| 214091_s_at | FPR1 | formyl peptide receptor 1 | 4.3 | 0 | Pro-inflammatory receptor | Chemotactic receptor |
| 206211_at | MME | membrane metallo-endopeptidase (neutral endopeptidase, enkephalinase, CALLA, CD10) | 4.3 | 8.6E-02 | Membrane metalloprotease | Can cleave angiotensin 1-9 |
| 216950_s_at | S100A7 | S100 calcium binding protein A7 (psoriasin 1) | 4.3 | 0 | S100 protein | Overexpressed during psoriasis |
| 227736_at | S100A12 | S100 calcium binding protein A12 (calgranulin C) | 4.2 | 0 | S100 protein, Antimicrobial | Possesses antifungal activity against *C. albicans* and is also active against *E. coli* and *P. aeruginosa* |
| 205916_at | AKR1B10 | aldo-keto reductase family 1, member B10 (aldose reductase) | 4.1 | 0 | Dual specificity phosphatase | Induced during oxidative stress |
| 226218_at | SPRR2B | small proline-rich protein 2B | 4.0 | 0 | Small proline rich protein | Cross-linked envelope protein of keratinocytes |
| 224917_at | APOBEC3A | apolipoprotein B mRNA editing enzyme, catalytic polypeptide-like 3A | 3.9 | 0 | anti-viral activity | Destroys foreign DNA through extensive deamination of cytosine |
| 206569_at | FOSL1 | FOS-like antigen 1 | 3.9 | 0 | Transcription factor | Part of transcription factor complex AP-1 |
| 210119_at | S100A8 | S100 calcium binding protein A8 (Calgranulin A) | 3.9 | 0 | S100, Antimicrobial peptide | Has antimicrobial activity towards bacteria and fungi |
| 203234_at | IL13RA2 | interleukin 13 receptor, alpha 2 | 3.9 | 0 | Decoy receptor | Lacks intracellular domain |
| 222162_s_at | TMPRSS11D | transmembrane protease, serine 11D | 3.8 | 5.9E-02 | Transmembrane protease | Involved in host defense systems on the mucous membrane |
| 205863_at | CXCL2 | chemokine (C-X-C motif) ligand 2 | 3.8 | 0 | Chemokine | Chemotactic for polymorphonuclear leukocytes and hematopoietic stem cells |
| 219554_at | FCGR1A | Fc fragment of IgG, high affinity Ia, receptor (CD64) | 3.8 | 0 | Fc-gamma receptor | High affinity receptor for the Fc region of immunoglobulins gamma |
| 210873_x_at | KRT6A | keratin 6A | 3.8 | 0 | Keratin | Forms intracellular intermediate filaments |
| 210164_at | TDO2 | tryptophan 2,3-dioxygenase | 3.7 | 2.2E-01 | Enzyme | Has antimicrobial, antiparasitic and antiviral effects |
| 202672_s_at | AREG | amphiregulin | 3.7 | 0 | Growth factor | EGF receptor ligand |
| 204475_at | TMPRSS4 | transmembrane protease, serine 4 | 3.7 | 0 | Transmembrane protease | Detaches keratinocytes from basement membrane |
| 224071_at | HBEGF | heparin-binding EGF-like growth factor | 3.6 | 5.6E-02 | Growth factor | EGF receptor ligand |
| 205207_at | SOCS3 | suppressor of cytokine signaling 3 | 3.6 | 0 | STAT-induced STAT inhibitor | Supresses cytokine signaling |
| 218960_at | UPP1 | uridine phosphorylase 1 | 3.5 | 0 | Phosporylase | Metabolism, nucleotide synthesis |
| 203936_s_at | KLK6 | kallikrein 6 | 3.5 | 0 | Serine protease | Sheds E-cadherin on keratinocytes |
| 214370_at | GPX3 | glutathione peroxidase 3 (plasma) | 3.4 | 0 | Peroxidase | Functions in the detoxification of hydrogen peroxide |
| 204733_at | CCL18 | chemokine (C-C motif) ligand 18 (pulmonary and activation-regulated) | 3.3 | 1.3E-01 | Chemokine | Associated with atopic dermatitis |
| 205680_at | SERPINA1 | serpin peptidase inhibitor, clade A (alpha-1 antiproteinase, antitrypsin), member 1 | 3.3 | 0 | Peptidase inhibitor | Inhibits elastase etc |
| 209125_at | MMP9 | matrix metallopeptidase 9 | 3.2 | 0 | Matrix metalloprotease | Keratinocyte migration |
| 227697_at | MIR21 | microRNA 21 | 3.2 | 0 | Micro RNA | Involved in cell cycle regulation |
| 202779_s_at | RHCG | Rh family, C glycoprotein | 3.2 | 0 | Ammonium transporter | May regulate transepithelial ammonia secretion |
| 216248_s_at | NR4A1 | nuclear receptor subfamily 4, group A | 3.2 | 1.1E-01 | Nuclear receptor | Transcription factor, Orphan nuclear receptor |
| 203764_at | PI15 | peptidase inhibitor 15 | 3.2 | 0 | Peptidase inhibitor | Trypsin inhibitor |
| 202628_s_at | S100P | S100 calcium binding protein P | 3.1 | 0 | S100 protein | Expressed in suprabasal layers of epithelial cells |
| 217497_at | SELL | selectin L (lymphocyte adhesion molecule 1) | 3.1 | 0 | Selectin | Cell-surface glycoprotein having a role in immunoadhesion |
| 239430_at | G0S2 | G0/G1switch 2 | 3.1 | 7.4E-02 | Cell cycle protein | Differentiation, Promotes apoptosis by binding to BCL2, upregulated in epidermolysis bullosa |
| 211429_s_at | IL7R | Interleukin 7 receptor | 3.1 | 0 | Receptor | Interleukin receptor |
| 228726_at | BCL2A1 | BCL2-related protein A1 | 3.1 | 1.4E-01 | BCL-2 family | Anti-apoptotic, reduces release of cytochrome c from mitochondria, blocks caspase activation |
| 229947_at | NR4A2 | nuclear receptor subfamily 4, group A, member 2 | 3.0 | 0 | Nuclear receptor | Transcription factor, Orphan nuclear receptor |
| 215049_x_at | GJB2 | gap junction protein, beta 2, 26kDa (connexin 26) | 3.0 | 0 | gap junction protein | Part of connexons |
| 1569003_at | CXCR4 | chemokine (C-X-C motif) receptor 4 | 3.0 | 0 | chemokine receptor | Important for lung epithelial migration in response to wounding |
| 205119_s_at | GPRC5A | G protein-coupled receptor, family C, group 5, member A | 3.0 | 7.4E-02 | type 3 G protein-coupling receptor | GPCR could modulate differentiation and maintaining homeostasis of epithelial cells. |
| 228729_at | DSC2 | desmocollin 2 | 2.9 | 0 | desmosomal family protein | Component of intercellular desmosome junctions |
| 204420_at | RGS1 | regulator of G-protein signaling 1 | 2.9 | 0 | Regulator of G-protein signalling | Inhibits signal transduction by increasing the GTPase activity of G protein alpha subunits |
| 205239_at | SERPINB1 | serpin peptidase inhibitor, clade B, member 1 | 2.9 | 0 | Peptidase inhibitor | Inhibigts neutrophil elastase, cathepsin G, and proteinase-3 and thus protects tissues from damage |
| 209774_x_at | KCNJ15 | potassium inwardly-rectifying channel, subfamily J, member 15 | 2.9 | 0 | potassium channel | Allows potassium to flow into a cell rather than out of a cell |
| 217028_at | CTLA4 | cytotoxic T-lymphocyte-associated protein 4 | 2.9 | 0 | Inhibitory receptor | Inhibitory receptor acting as a major negative regulator of T-cell responses |
| 227458_at | DLGAP5 | discs, large homolog 7 (Drosophila) | 2.8 | 0 | Mitotic phosphoprotein | Key regulator of adherens junction integrity and differentiation |
| 226535_at | ATF3 | activating transcription factor 3 | 2.8 | 0 | Transcription factor (CREB family) | Represses transcription from promoters with ATF sites, involved in cellular stress responses |
| 236341_at | CHI3L2 | chitinase 3-like 2 | 2.8 | 1.3E-01 | Non-functional chitinase | Similar to bacterial chitinases but lacks chitinase activity, involved in cartilage biogenesis |
| 204563_at | RRM2 | ribonucleotide reductase M2 polypeptide | 2.8 | 0 | Ribonucleotide reductase | Catalyzes the biosynthesis of deoxyribonucleotides from the corresponding ribonucleotides. |
| 216834_at | TFPI2 | tissue factor pathway inhibitor 2 | 2.8 | 1.2E-01 | Proteinase inhibitor | May regulated plasmin-mediated matrix remodeling. Inhibits trypsin, plasmin, factor VIIa/tissue factor |
| 207602_at | C10orf99 | chromosome 10 open reading frame 99 | 2.7 | 0 | uncharacterized protein | Unknown function |
| 205513_at | GPR183 | Epstein-Barr virus induced gene 2 (lymphocyte-specific G protein-coupled receptor) | 2.7 | 9.5E-02 | Receptor | Unknown function |
| 221601_s_at | CARHSP1 | calcium regulated heat stable protein 1, 24kDa | 2.6 | 0 | Heat stable protein | Binds mRNA and regulates the stability of target mRNA. |
| 38037_at | FAIM3 | Fas apoptotic inhibitory molecule 3 | 2.6 | 5.6E-02 | Receptor | Antiapoptotic, Protects cells from FAS-, TNF alpha- and FADD-induced apoptosis, Fc receptor for IgM |
| 202340_x_at | CD274 | CD274 molecule (programmed cell death 1 ligand 1) | 2.6 | 0 | Receptor ligand | Ligand for PD-1 receptor on CD8+ T cells. Transmits inhibitory signal to reduces proliferation of T-cells |
| 216615_s_at | MMP12 | matrix metallopeptidase 12 (macrophage elastase) | 2.6 | 7.7E-02 | Matrix metalloprotease | Degrades soluble and insoluble elastin, involved in tissue injury and remodeling |
| 205419_at | VMP1 | vacuole membrane protein 1 (TMEM49) | 2.6 | 0 | Stress-induced protein | Formation of cell junctions, involved in autophagy, contains MIR21 within the gene sequence |
| 205428_s_at | HTR3A | 5-hydroxytryptamine (serotonin) receptor 3A | 2.6 | 1.0E-01 | ligand-gated ion channel receptor | Receptor for serotonin |
| 203434_s_at | UBE2S | ubiquitin-conjugating enzyme E2S | 2.5 | 0 | ubiquitin-conjugating enzyme | Ubiquitination and subsequent degradation of VHL, resulting in an accumulation of HIF1A. |
| 204580_at | ITGB6 | integrin, beta 6 | 2.5 | 0 | Integrin | receptor for fibronectin and cytotactin, highly expressed in poorly healing human wounds |
| 203108_at | TYMP | endothelial cell growth factor 1 (platelet-derived) | 2.5 | 0 | angiogenic factor | Angiogenic activity and chemotactic activity Catalyzes reversible phosphorolysis of thymidine. |
| 213524_s_at | CALB2 | calbindin 2, 29kDa (calretinin) | 2.5 | 9.2E-02 | intracellular calcium-binding protein | Intracellular calcium buffering |
| 211506_s_at | SELE | selectin E (endothelial adhesion molecule 1) | 2.5 | 0 | Selectin | Cell-surface glycoprotein having a role in immunoadhesion |
| 205681_at | DSG3 | desmoglein 3 (pemphigus vulgaris antigen) | 2.4 | 0 | Desmoglein family member | Component of desmosomes, expressed in proliferating basal layers of epidermis |
| 209924_at | ADAMTS1 | ADAM metallopeptidase with thrombospondin type 1 motif, 1 | 2.4 | 0 | ADAMTS protein family member | Cleaves aggrecan, may be involved in keratinocyte differentiation and migration during wound healing |
| 213060_s_at | CCNB1 | cyclin B1 | 2.4 | 0 | Regulatory protein involved in mitosis | Essential for the control of the cell cycle at the G2/M (mitosis) transition |
| 209278_s_at | S100A2 | S100 calcium binding protein A2 | 2.4 | 0 | S100 protein, | Toumor supressor, involved in keratinocyte differentiation |
| 205943_at | CD163 | CD163 molecule | 2.4 | 0 | Scavenger receptor | Recognizes gram positive and gram negative bacteria |
| **Downregulated Genes** | | | | | | |
| 218876_at | ELN | elastin | -4.5 | 9.8E-02 | Extracellular matrix protein | Inhibits terminal differentiation of keratinocytes |
| 239183_at | WIF1 | WNT inhibitory factor 1 | -3.5 | 1.6E-01 | Signal inhibitor | Inhibits WNT proteins and WNT signalling. Tumor supressor |
| 203824_at | OMD | osteomodulin | -3.2 | 8.1E-02 | Proteoglycan, Extracellular matrix protein | May be involved in mineralization process |
| 228241_at | ANGPTL1 | angiopoietin-like 1 | -3.1 | 0 | VEGF family member | Unknown function |
| 212670_at | TNN | tenascin N | -3.0 | 2.0E-01 | Extracellular matrix glycoprotein | May inhibit neuronal outgrowth |
| 239349_at | BTC | betacellulin | -2.8 | 7.7E-02 | Growth factor, member of the EGF family | Ligand for the EGF receptor |
| 205907_s_at | AGR3 | breast cancer membrane protein 11 | -2.6 | 0 | Membrane protein | Highly upregulated in ovarian tumors |
| 241412_at | TSPAN8 | tetraspanin 8 | -2.5 | 0 | Cell surface glycoprotein | Is known to complex with integrins, may mediate signal transduction events that play a role in the regulation of cell development, activation, growth and motility |
| 205200_at | CLEC3B | C-type lectin domain family 3, member B (tetranectin) | -2.4 | 1.6E-01 | C-type lectin | Binds to plasminogen and enhances plasminogen activation. Wound healing is delayed in tetranectin-null mice |
| 204712_at | TNXA | tenascin XA | -2.4 | 2.5E-01 | Pseudogene |  |
| 215271_at | C1QTNF7 | C1q and tumor necrosis factor related protein 7 | -2.4 | 8.6E-02 | Receptor ligand | Unknown function |
| 216333_x_at | TPPP3 | tubulin polymerization-promoting protein family member 3 | -2.4 | 0 | Tubulin polymerization-promoting protein | Binds tubulin and has microtubule bundling activity. May play a role in cell proliferation and mitosis |
