# Supplementary material for: Transcription Factor Binding Site Analysis Identifies FOXO Transcription Factors as Regulators of the Cutaneous Wound Healing Process
Source: PLoS One. 2014 Feb 19;9(2):e89274. doi: 10.1371/journal.pone.0089274 (PMC3929751; doi:10.1371/journal.pone.0089274)
Supplement: Table S2 — List of the 100 most enriched transcription factor binding sites in the promoter regions of the 100 most differentially expressed genes between wounded and non-wounded skin during the proliferative phase of wound healing. The list of 100 genes used for SMART analysis (see Table S1) was acquired by a complete re-analysis of the data set previously published by Roupé et al 2009 (see Materials and Methods). FOXO transcription factors have been highlighted in yellow. (DOCX) [file pone.0089274.s003.docx]

**Supplementary Table 2**

List of the 100 most enriched transcription factor binding sites in the promoter regions of the 100 most differentially expressed genes between wounded and non-wounded skin during the proliferative phase of wound healing. The list of 100 genes used for analysis was acquired by a complete re-analysis of the data set previously published by Roupé et al 2009 (see Materials and Methods).

| **Transcription factor** | **No. of hits** | **Significant Enrichment (PE)** | **% of motif presence in no. of promoters** |
| --- | --- | --- | --- |
| FOXO4 | 720 | 1.00E-05 | 100 |
| FOXO1 | 728 | 1.00E-05 | 100 |
| BCL6 | 1382 | 1.00E-05 | 100 |
| STAT5A | 1774 | 1.00E-05 | 100 |
| SRY | 959 | 1.00E-05 | 100 |
| STAT6 | 1375 | 1.00E-05 | 100 |
| AP1 | 2450 | 1.00E-05 | 98.97959 |
| STAT4 | 599 | 1.00E-05 | 96.938774 |
| HNF3 | 777 | 1.00E-05 | 96.938774 |
| CEBPA | 160 | 1.00E-05 | 74.4898 |
| ZTA | 92 | 1.00E-05 | 59.183674 |
| GEN | 1877 | 2.00E-05 | 100 |
| ELF1 | 307 | 2.00E-05 | 94.89796 |
| HMGIY | 944 | 3.00E-05 | 100 |
| FAC1 | 80 | 1.00E-04 | 55.10204 |
| IPF1 | 348 | 1.50E-04 | 84.69388 |
| AIRE | 27 | 1.50E-04 | 20.408163 |
| HNF3B | 250 | 4.90E-04 | 80.61224 |
| HFH3 | 172 | 5.80E-04 | 75.5102 |
| HNF3ALPHA | 200 | 6.40E-04 | 81.63265 |
| CDXA | 1200 | 6.60E-04 | 97.95918 |
| GFI1 | 602 | 7.10E-04 | 94.89796 |
| NFAT | 1465 | 7.50E-04 | 100 |
| LEF1 | 850 | 9.50E-04 | 100 |
| CEBP | 800 | 9.70E-04 | 100 |
| BRN2 | 181 | 0.00108 | 74.4898 |
| BACH2 | 45 | 0.00127 | 37.7551 |
| FOXJ2 | 161 | 0.00148 | 73.46939 |
| SOX17 | 344 | 0.00153 | 87.755104 |
| IRF | 163 | 0.00157 | 50 |
| TBP | 1039 | 0.0016 | 94.89796 |
| CIZ | 870 | 0.0021 | 95.918365 |
| MTATA | 65 | 0.00226 | 47.959183 |
| TATA | 378 | 0.00238 | 86.734695 |
| GR | 745 | 0.00246 | 94.89796 |
| ETS | 1281 | 0.00248 | 100 |
| XVENT1 | 120 | 0.00251 | 62.2449 |
| CEBPGAMMA | 27 | 0.00321 | 23.469387 |
| TCF11 | 265 | 0.00334 | 91.83673 |
| IRF1 | 60 | 0.0039 | 40.816326 |
| MEIS1 | 383 | 0.00395 | 98.97959 |
| SOX9 | 202 | 0.00404 | 81.63265 |
| LPOLYA | 275 | 0.0041 | 84.69388 |
| FREAC2 | 102 | 0.00416 | 50 |
| LDSPOLYA | 83 | 0.00454 | 57.142857 |
| RBPJK | 274 | 0.00476 | 87.755104 |
| TEF | 19 | 0.005 | 14.285714 |
| PEA3 | 668 | 0.00567 | 100 |
| ETS1 | 197 | 0.00608 | 82.65306 |
| CEBPDELTA | 88 | 0.00694 | 56.122448 |
| DBP | 467 | 0.00718 | 96.938774 |
| MAF | 84 | 0.00723 | 60.204082 |
| CAP | 406 | 0.00793 | 98.97959 |
| PAX2 | 509 | 0.00823 | 97.95918 |
| XFD1 | 52 | 0.0092 | 43.877552 |
| TFE | 117 | 0.00957 | 64.28571 |
| PU1 | 365 | 0.01076 | 90.81633 |
| FREAC7 | 224 | 0.01089 | 76.53061 |
| NKX25 | 723 | 0.01109 | 96.938774 |
| POU6F1 | 7 | 0.01283 | 7.142857 |
| GATA1 | 1919 | 0.0144 | 100 |
| BLIMP1 | 22 | 0.01546 | 22.44898 |
| SOX5 | 107 | 0.01585 | 65.30612 |
| AR | 497 | 0.01696 | 98.97959 |
| TAL1ALPHAE47 | 53 | 0.01791 | 37.7551 |
| STAT | 164 | 0.01909 | 74.4898 |
| NKX61 | 29 | 0.01987 | 23.469387 |
| AREB6 | 1745 | 0.02012 | 100 |
| FOXO3 | 188 | 0.02122 | 75.5102 |
| E4BP4 | 14 | 0.02214 | 12.244898 |
| PR | 480 | 0.02277 | 94.89796 |
| RUSH1A | 235 | 0.02335 | 79.591835 |
| NFE2 | 17 | 0.02377 | 15.306123 |
| DTYPEPA | 9 | 0.02398 | 9.183674 |
| LMO2COM | 522 | 0.02436 | 100 |
| LEF1TCF1 | 204 | 0.02441 | 87.755104 |
| TEF1 | 122 | 0.02584 | 72.44898 |
| OG2 | 472 | 0.02619 | 88.77551 |
| S8 | 454 | 0.02687 | 88.77551 |
| BACH1 | 20 | 0.02692 | 18.367348 |
| NKX62 | 446 | 0.03088 | 88.77551 |
| SMAD | 904 | 0.03199 | 100 |
| GATA2 | 518 | 0.03266 | 94.89796 |
| SOX10 | 597 | 0.03297 | 96.938774 |
| TAL1BETAE47 | 55 | 0.03396 | 39.795918 |
| HNF1 | 47 | 0.03425 | 22.44898 |
| NKX3A | 64 | 0.03475 | 37.7551 |
| GFI1B | 37 | 0.03478 | 30.612246 |
| TFIIA | 25 | 0.03559 | 21.428572 |
| HLF | 16 | 0.0371 | 15.306123 |
| XFD2 | 73 | 0.03779 | 52.040817 |
| GATA3 | 465 | 0.03784 | 95.918365 |
| NKX32 | 492 | 0.03789 | 98.97959 |
| HNF6 | 49 | 0.03795 | 40.816326 |
| CHOP | 58 | 0.03806 | 45.918365 |
| CDX | 34 | 0.03974 | 28.571428 |
| HFH1 | 48 | 0.04116 | 32.65306 |
| TGIF | 179 | 0.04254 | 83.67347 |
| XPF1 | 51 | 0.04265 | 41.836735 |
