# Supplementary material for: Transcription Factor Binding Site Analysis Identifies FOXO Transcription Factors as Regulators of the Cutaneous Wound Healing Process
Source: PLoS One. 2014 Feb 19;9(2):e89274. doi: 10.1371/journal.pone.0089274 (PMC3929751; doi:10.1371/journal.pone.0089274)
Supplement: Table S6 — The 100 most differentially expressed genes between wounded and non-wounded skin during the acute phase of wound healing adapted from the list published by Kennedy-Crispin et al 2011. Since the list from Kennedy-Crispin et al 2011 originally contained several duplicates and 2 genes also were excluded from analysis the resulting list used for analysis contained the 88 genes in the table. Genes with increased expression are colored red and genes with decreased expression are colored blue. The 46 genes containing co-occurring FOXO1, FOXO4 and FOXO3 transcription factor binding sites are marked with *. (DOCX) [file pone.0089274.s007.docx]

**Supplementary Table 6**

The list of genes is adapted from the list published by Kennedy-Crispin et al 2011. Since it originally contained several duplicates and 2 genes also were excluded from analysis the resulting list used for analysis contained the 88 genes listed below. Genes with increased expression are colored red and genes with decreased expression are colored blue. The 46 genes containing co-occurring FOXO1, FOXO4 and FOXO3 transcription factor binding sites are marked with *.

| **Gene symbol** | **Description** |
| --- | --- |
| DSC2 | desmocollin 2 |
| LIPG* | lipase, endothelial |
| ZBTB10* | zinc finger and BTB domain containing 10 |
| GABPB1* | GA binding protein transcription factor, beta subunit 1 |
| SOX9 | SRY-box 9 |
| IQCG* | IQ motif containing G |
| UPP1 | uridine phosphorylase 1 |
| CAMK2N1* | calcium/calmodulin-dependent protein kinase II inhibitor 1 |
| GADD45B | growth arrest and DNA-damage-inducible, beta |
| CXCL3* | chemokine (C-X-C motif) ligand 3 |
| DUSP10 | dual specificity phosphatase 10 |
| PTP4A1 | Protein tyrosine phosphatase type IVA, member 1 |
| CXCL2* | chemokine (C-X-C motif) ligand 2 |
| SOD2* | Superoxide dismutase 2, mitochondrial |
| SERPINB4* | serpin peptidase inhibitor, clade B (ovalbumin), member 4 |
| BHLHE40 | basic helix-loop-helix family, member e40 |
| HS3ST1* | heparan sulfate (glucosamine) 3-O-sulfotransferase 1 |
| ARL14* | ADP-ribosylation factor-like 14 |
| EGR4 | early growth response 4 |
| INHBA | inhibin, beta A (activin A, activin AB alpha polypeptide) |
| TGFB2* | Transforming growth factor, beta 2 |
| CXCR4 | chemokine (C-X-C motif) receptor 4 |
| FOSL1 | FOS-like antigen 1 |
| GPRC5A* | G protein-coupled receptor, family C, group 5, member A |
| IL13RA2 | interleukin 13 receptor, alpha 2 |
| PLAUR* | plasminogen activator, urokinase receptor |
| RHCG* | Rh family, C glycoprotein |
| NEDD4L* | Neural precursor cell expressed, developmentally down-regulated 4-like |
| ALDH1A3 | aldehyde dehydrogenase 1 family, member A3 |
| IL1RL1 | interleukin 1 receptor-like 1 |
| MMP1* | matrix metallopeptidase 1 (interstitial collagenase) |
| IL8* | interleukin 8 |
| CCL20* | chemokine (C-C motif) ligand 20 |
| MMP3* | matrix metallopeptidase 3 (stromelysin 1, progelatinase) |
| APOBEC3A | apolipoprotein B mRNA editing enzyme, catalytic polypeptide-like 3A |
| PHLDA1* | pleckstrin homology-like domain, family A, member 1 |
| KRT6B* | keratin 6B |
| ISG20* | interferon stimulated exonuclease gene 20kDa |
| PPP1R15A | protein phosphatase 1, regulatory (inhibitor) subunit 15A |
| ZNF165* | zinc finger protein 165 |
| PDE4B* | phosphodiesterase 4B, cAMP-specific |
| AREG | Amphiregulin (schwannoma-derived growth factor) |
| HBEGF | heparin-binding EGF-like growth factor |
| HSPA6 | heat shock 70kDa protein 6 (HSP70B') |
| DHRS9* | dehydrogenase/reductase (SDR family) member 9 |
| KRT6A* | Keratin 6A |
| S100A9 | S100 calcium binding protein A9 (calgranulin B) |
| PMAIP1* | phorbol-12-myristate-13-acetate-induced protein 1 |
| ATF3 | activating transcription factor 3 |
| JUN* | v-jun sarcoma virus 17 oncogene homolog (avian) |
| TNFAIP3 | tumor necrosis factor, alpha-induced protein 3 |
| APOBEC3B | apolipoprotein B mRNA editing enzyme, catalytic polypeptide-like 3B |
| TM4SF1* | transmembrane 4 L six family member 1 |
| S100A8* | S100 calcium binding protein A8 (calgranulin A) |
| FCGBP | Fc fragment of IgG binding protein |
| ALOX5 | arachidonate 5-lipoxygenase |
| CMAH | cytidine monophosphate-N-acetylneuraminic acid hydroxylase |
| CD1A* | CD1a molecule |
| CD207 | CD207 molecule, langerin |
| FCER1A* | Fc fragment of IgE, high affinity I, receptor for; alpha polypeptide |
| NHLH2* | nescient helix loop helix 2 |
| GPLD1* | glycosylphosphatidylinositol specific phospholipase D1 |
| ANK3* | ankyrin 3, node of Ranvier (ankyrin G) |
| COL21A1 | collagen, type XXI, alpha 1 /// collagen, type XXI, alpha 1 |
| C14orf132 | chromosome 14 open reading frame 132 |
| FRY | furry homolog (Drosophila) |
| ZNF652 | zinc finger protein 652 |
| EFHC2* | EF-hand domain (C-terminal) containing 2 |
| UNC93A* | unc-93 homolog A (C. elegans) |
| ANXA9 | annexin A9 |
| C1orf68* | chromosome 1 open reading frame 68 |
| SLURP1 | secreted LY6/PLAUR domain containing 1 |
| LAMB4* | laminin, beta 4 |
| ASPN | asporin (LRR class 1) |
| BCL11A | B-cell CLL/lymphoma 11A (zinc finger protein) |
| EXPH5* | Exophilin 5 |
| SLC16A7* | solute carrier family 16, member 7 (monocarboxylic acid transporter 2) |
| HLA-DPB1 | major histocompatibility complex, class II, DP beta 1 |
| CTSH | cathepsin H |
| MXRA5 | matrix-remodelling associated 5 |
| AZGP1 | alpha-2-glycoprotein 1, zinc |
| LCE2B* | late cornified envelope 2B |
| HLA-DPA1* | major histocompatibility complex, class II, DP alpha 1 |
| ARG1 | arginase, liver |
| HOXA9 | homeobox A9 |
| CDHR1 | cadherin-related family member 1 |
| FLG* | filaggrin |
| LOR* | loricrin |
