# Supplementary material for: Transcription Factor Binding Site Analysis Identifies FOXO Transcription Factors as Regulators of the Cutaneous Wound Healing Process
Source: PLoS One. 2014 Feb 19;9(2):e89274. doi: 10.1371/journal.pone.0089274 (PMC3929751; doi:10.1371/journal.pone.0089274)
Supplement: Table S7 — List of the 100 most enriched transcription factor binding sites in the promoter regions of the most differentially expressed genes between wounded and non-wounded epidermis published by Kennedy-Crispin et al 2011. FOXO transcription factors have been highlighted in yellow. (DOCX) [file pone.0089274.s008.docx]

**Supplementary Table S7**

List of the 100 most enriched transcription factor binding sites in the promoter regions of the most differentially expressed genes between wounded and non-wounded epidermis published by Kennedy-Crispin et al 2011.

| **Transcription factor** | **No. of hits** | **Significant Enrichment (PE)** | **% of motif presence in no. of promoters** |
| --- | --- | --- | --- |
| AP1 | 1927 | 1.00E-04 | 97.53086 |
| FOXO4 | 566 | 1.90E-04 | 97.53086 |
| FOXO1 | 566 | 2.10E-04 | 97.53086 |
| FAC1 | 67 | 2.90E-04 | 54.320988 |
| SRY | 748 | 3.10E-04 | 100 |
| ZTA | 68 | 5.20E-04 | 55.555557 |
| XPF1 | 53 | 6.10E-04 | 50.617283 |
| AP3 | 122 | 7.30E-04 | 69.1358 |
| HNF3ALPHA | 169 | 8.40E-04 | 75.30864 |
| BCL6 | 1056 | 8.60E-04 | 100 |
| CDXA | 1000 | 0.0012 | 98.765434 |
| GR | 633 | 0.00145 | 96.296295 |
| HNF3 | 595 | 0.00164 | 90.12346 |
| STAT5A | 1327 | 0.00246 | 100 |
| MAF | 74 | 0.00305 | 61.728394 |
| HFH3 | 138 | 0.00377 | 69.1358 |
| TBP | 850 | 0.00382 | 96.296295 |
| P53 | 162 | 0.00426 | 77.77778 |
| PR | 421 | 0.00426 | 96.296295 |
| STAT4 | 441 | 0.0044 | 97.53086 |
| XVENT1 | 99 | 0.00481 | 56.790123 |
| HLF | 17 | 0.00483 | 19.753086 |
| SOX9 | 170 | 0.00485 | 85.18519 |
| FREAC2 | 86 | 0.00516 | 61.728394 |
| HNF3B | 197 | 0.0052 | 77.77778 |
| GEN | 1447 | 0.00537 | 100 |
| HFH4 | 23 | 0.00736 | 24.691359 |
| GATA | 461 | 0.00812 | 92.59259 |
| GATA6 | 230 | 0.00848 | 92.59259 |
| RUSH1A | 204 | 0.00957 | 83.950615 |
| POU1F1 | 52 | 0.00969 | 49.382717 |
| BACH2 | 33 | 0.01003 | 33.333332 |
| NKX22 | 32 | 0.01103 | 27.160494 |
| AIRE | 18 | 0.01276 | 18.518518 |
| HFH8 | 84 | 0.01289 | 56.790123 |
| SOX17 | 273 | 0.01335 | 95.06173 |
| TFE | 96 | 0.01384 | 59.25926 |
| CART1 | 21 | 0.01465 | 22.222221 |
| DBP | 385 | 0.01494 | 98.765434 |
| LPOLYA | 222 | 0.01567 | 83.950615 |
| STAT6 | 1048 | 0.01604 | 100 |
| BRCA | 341 | 0.01605 | 96.296295 |
| HNF6 | 44 | 0.01622 | 38.271606 |
| LDSPOLYA | 65 | 0.0176 | 54.320988 |
| FREAC7 | 184 | 0.01825 | 80.24691 |
| LEF1TCF1 | 173 | 0.01844 | 83.950615 |
| XFD3 | 23 | 0.02062 | 24.691359 |
| ROAZ | 2 | 0.02113 | 2.4691358 |
| GCNF | 3 | 0.02138 | 3.7037036 |
| FREAC4 | 11 | 0.02341 | 12.345679 |
| MTATA | 49 | 0.02457 | 45.679012 |
| XFD1 | 42 | 0.02471 | 43.209877 |
| LEF1 | 672 | 0.0248 | 100 |
| NKX25 | 593 | 0.02617 | 97.53086 |
| ETS1 | 158 | 0.02889 | 88.888885 |
| BACH1 | 17 | 0.02984 | 18.518518 |
| GATA1 | 1577 | 0.03079 | 100 |
| CDPCR3 | 3 | 0.03233 | 3.7037036 |
| SOX5 | 87 | 0.03406 | 64.19753 |
| FOXJ2 | 122 | 0.03414 | 66.666664 |
| HFH1 | 41 | 0.03792 | 41.975307 |
| IPF1 | 251 | 0.03837 | 83.950615 |
| CEBPB | 131 | 0.03994 | 72.83951 |
| MMEF2 | 57 | 0.03994 | 46.91358 |
| TEF1 | 100 | 0.04279 | 69.1358 |
| FOXM1 | 72 | 0.04327 | 49.382717 |
| TFIIA | 21 | 0.04358 | 22.222221 |
| MSX1 | 31 | 0.04961 | 29.62963 |
| CEBP | 623 | 0.05385 | 100 |
| GATA2 | 425 | 0.0549 | 96.296295 |
| POU6F1 | 5 | 0.05509 | 6.1728396 |
| NFAT | 1139 | 0.05558 | 100 |
| OG2 | 384 | 0.05624 | 88.888885 |
| PXRRXR | 271 | 0.05635 | 83.950615 |
| MEIS1 | 300 | 0.0566 | 97.53086 |
| PAX2 | 405 | 0.0571 | 93.82716 |
| TAL1BETAE47 | 45 | 0.05752 | 44.444443 |
| TATA | 263 | 0.05755 | 81.48148 |
| S8 | 369 | 0.05909 | 88.888885 |
| KAISO | 102 | 0.06064 | 69.1358 |
| SMAD | 742 | 0.06077 | 100 |
| GRE | 4 | 0.06097 | 4.9382715 |
| RBPJK | 211 | 0.06103 | 87.65432 |
| SOX10 | 489 | 0.06179 | 96.296295 |
| NKX62 | 363 | 0.06234 | 88.888885 |
| GFI1B | 30 | 0.06291 | 29.62963 |
| CHX10 | 33 | 0.06365 | 29.62963 |
| XFD2 | 59 | 0.06499 | 51.851852 |
| HMGIY | 706 | 0.06794 | 100 |
| CEBPA | 105 | 0.07179 | 66.666664 |
| TAL1ALPHAE47 | 41 | 0.07331 | 39.506172 |
| IRF | 114 | 0.07431 | 49.382717 |
| FOXD3 | 50 | 0.08827 | 40.74074 |
| ELF1 | 204 | 0.0897 | 87.65432 |
| MYB | 974 | 0.09114 | 97.53086 |
| TCF4 | 99 | 0.09133 | 72.83951 |
| FOXO3 | 148 | 0.09215 | 81.48148 |
| TST1 | 38 | 0.09462 | 30.864197 |
